# Decoding substrate recognition in malapain-2 through structural and mutational insights

**DOI:** 10.1016/j.csbj.2025.10.043

**Published:** 2025-10-24

**Authors:** Sian D’silva, Hương Giang Lȇ, Byoung-Kuk Na, Soumyananda Chakraborti

**Affiliations:** a Department of Biological Science, Birla Institute of Technology and Sciences-Pilani (Hyderabad campus), Hyderabad, India; b Department of Parasitology and Tropical Medicine, Department of Convergence Medical Science, and Institute of Medical Science, Gyeongsang National University College of Medicine, Jinju 52727, South Korea

**Keywords:** *Plasmodium malariae*, Cysteine proteases, Malapain-2, Haemoglobin degradation, Substrate specificity, Site-directed mutagenesis, Enzyme kinetics, Structural bioinformatics, Antimalarial drug targets

## Abstract

Cysteine proteases of the falcipain (FP) family are essential for the survival and pathogenicity of *Plasmodium* parasites and represent promising targets for antimalarial drug development. These enzymes mediate haemoglobin degradation during the intraerythrocytic stage, providing nutrients and facilitating parasite growth. While FP-family proteases from *P. falciparum* (e.g., FP-2A and FP-3) are well-characterized, their orthologs in less-studied species like *P. malariae* remain poorly understood. Given the rising concern over drug-resistant malaria and mixed-species infections, targeting diverse FP-family ortholog enzymes in other human malaria parasites is a timely and promising therapeutic strategy. In this study, we investigated malapain-2 (MP-2), a cysteine protease from *P. malariae* belonging to the FP-2A/FP-3 subfamily, with the aim of characterizing its substrate specificity and structural features in comparison to FP-2A. Using biochemical assays, we found that MP-2 exhibited a distinct substrate preference, favouring arginine at the P2 position, unlike FP-2A which prefers hydrophobic residues. Site-directed mutagenesis and structural modelling revealed that differences in the S2 substrate-binding sub-pocket (key protease site) account for this specificity shift. Key mutations in MP-2 that mimic FP-2A residues altered substrate preference, confirming the role of specific residues in substrate accommodation. Docking and molecular dynamics simulations further supported these findings by revealing altered interaction networks within the binding pocket. Our results highlight the unique enzymatic properties of MP-2 and its potential as a species-specific drug target. This work expands our understanding of FP-family proteases across *Plasmodium* species and provides a foundation for the rational design of broad-spectrum or species-selective cysteine protease inhibitors.

## Graphical Abstract

**Figure:**
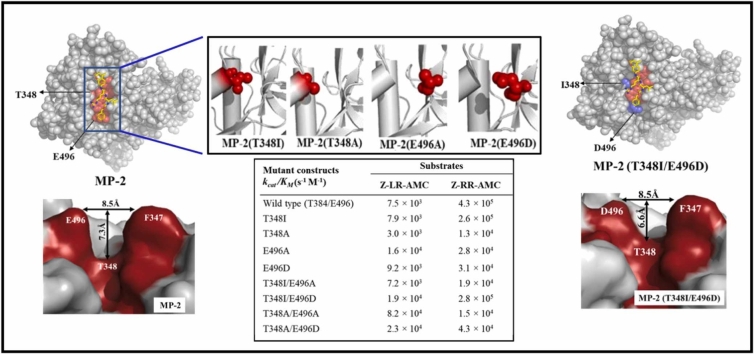

## 1 Introduction

According to the *World Malaria Report 2024*, global malaria cases rose to an estimated 263 million in 2023, up from 249 million in 2022, with a case incidence of 60.4 per 1,000 population at risk. Deaths slightly declined to 597,000, though sub-Saharan Africa continued to be the hotspot of malaria accounting for 94 % of cases and 95 % of deaths, mostly among children under five [1]. Nigeria, the Democratic Republic of the Congo, Niger, and Tanzania collectively contributed to over half of all malaria deaths [2]. While *Plasmodium falciparum* and *P. vivax* are the most widespread species, *P. malariae* also contributes to the global malaria burden, particularly as a cause of non-falciparum infections. A pooled analysis from 2000 to 2020 estimated a global prevalence of 2.01 % for *P. malariae*, with notable endemicity in sub-Saharan Africa especially in Nigeria, the Democratic Republic of the Congo, and Tanzania, where it commonly co-infects with *P. falciparum* [3]. Outside Africa, cases have been documented in India, Indonesia, Vietnam and parts of South America, with localized prevalence reaching 10–20 % in some asymptomatic populations [4], [5], [6], [7]. Due to its typically mild symptoms and ability to persist at low parasitaemia for extended periods, *P. malariae* infections are frequently underdiagnosed, contributing to chronic infections and ongoing transmission; unexpected outbreaks such as the one in Khanh Vinh District, Vietnam highlight the species’ capacity to re-emerge and cause substantial disease burden despite its historically low profile [7], [8].

*Plasmodium* proteases play a critical role in the parasite’s life cycle and represent promising drug targets for malaria treatment. These enzymes are involved in essential processes such as haemoglobin degradation, and host cell invasion and egression all vital for *Plasmodium* survival and pathogenicity [9], [10]. Among these, cysteine proteases like falcipains (FPs) facilitate haemoglobin digestion in the parasite’s acidic food vacuole, providing essential amino acids and creating space for parasite growth [11]. Other protease classes, including serine and aspartic proteases, are also key players in red blood cell invasion and immune evasion [12]. Targeting these proteases with specific inhibitors has shown considerable potential to impair parasite development and proliferation, making them attractive candidates for antimalarial drug development [13], [14]. Inhibitors directed at these enzymes offer a promising strategy to overcome resistance to current treatments by exploiting pathways essential to parasite viability, thereby opening new avenues to combat drug-resistant malaria strains [15], [16], [17], [18], [19].

Among the various protease targets, cysteine proteases particularly the FPs stand out for their essential roles in parasite survival and their potential as selective drug targets [20], [21], [22]. Beyond haemoglobin digestion, FPs are involved in erythrocyte invasion, egress, and gametocyte development, underscoring their multifunctionality. Their structural divergence from human cysteine proteases enhances the feasibility of selective inhibition. Indeed, inhibitors of FP activity disrupt haemoglobin degradation, resulting in parasite growth arrest and death.

Progress in FP research has yielded several promising inhibitors, reinforcing the viability of these proteases as drug targets [23], [24]. Structural and functional characterization of FPs is crucial for designing selective inhibitors that address both current treatment gaps and the looming threat of antimalarial drug resistance. Furthermore, mixed-species infections necessitate the development of broad-spectrum inhibitors targeting orthologous enzymes in other human malaria parasites, such as vivapains (*P. vivax*; VXs), knowpains (*P. knowlesi*; KPs), and malapains (*P. malariae*; MPs) [25], [26], [27], [28]

*P. falciparum* encodes four papain-like cysteine proteases FP-1, FP-2A, FP-2B, and FP-3 [29]. FP-2A, has a well-defined active site located within a surface depression characteristic of papain-family protease [30]. Its substrate specificity is largely determined by the S2 sub-pocket, a hydrophobic cavity (key site for protease action) formed by residues such as Trp-43, Leu-84, Ile-85, Ser-149, and Ala-175 [31]. A distinctive Asp-234 residue at the base of the S2 sub-pocket impairs the binding of positively charged residues like Arg and Lys, setting FP-2A apart from homologous proteases such as cruzipain or cathepsin B [31]. Moreover, a haemoglobin-binding β-hairpin motif near the C-terminus enhances substrate recognition, aligning with FP-2’s preference for hydrophobic residues at the P2 position [32]. These structural features offer critical leverage points for designing potent, selective inhibitors.

The emergence of chloroquine-resistant *P. malariae* strains in regions such as Indonesia has intensified the need for new therapeutic strategies [33], [34]. Despite their potential relevance, FP-family enzymes in *P. malariae* have remained largely uncharacterized. Recent studies have identified two key cysteine proteases, malapain-2 (MP-2) and malapain-4 (MP-4) belonging to the FP-2/FP-3 subfamily [26]. MP-2 and MP-4 have been identified as the principal food vacuole in *P. malariae* based on their biochemical properties closely resembling those of the FP-2A and FP-3 proteases. These enzymes effectively hydrolyse haemoglobin and dipeptidyl substrates in acidic conditions consistent with the food vacuole environment, supporting their critical roles in nutrient acquisition. Beyond haemoglobin digestion, MPs can degrade cytoskeletal proteins such as spectrin and band 3 present in red blood cells, implicating them in parasite egress and remodelling of host erythrocytes [26]. Physicochemical analyses show that MP-2 and MP-4 have molecular weights around 27 kDa, acidic isoelectric points (~4.6–4.8), and negative hydrophobicity, reflecting their hydrophilic, soluble nature, appropriate for intracellular enzymatic activity. Our computational structural modelling confirmed that MP-2 and related proteins adopt the characteristic papain-like fold of cysteine proteases, consistent with strong structural conservation within this enzyme family.

Despite sharing structural similarities with their FP counterparts, they exhibit distinct substrate preferences. Notably, MP-2 shows a marked preference for arginine at the P2 position, whereas MP-4 favours leucine and also accommodates phenylalanine. Both proteases possess the conserved catalytic triad and specialized substrate-binding pockets crucial for their enzymatic function. These differences, along with MP-2’s conserved catalytic triad and specialized binding pocket, highlight its potential as a selective drug target. Both MP-2 and MP-4 function optimally at pH 5.0 and remain active across a broad pH range, consistent with their roles in haemoglobin degradation and erythrocyte remodelling.

In this study, we thoroughly investigated the substrate specificity of MP-2 and its divergence from FP-2A by integrating protein engineering with advanced computational techniques. While FPs have been extensively characterized and validated as antimalarial drug targets, the structural and functional properties of MPs remain largely uncharacterized. Utilizing site-directed mutagenesis, we identified key molecular determinants underlying MP-2’s unique substrate preference and engineered targeted mutations to interchange critical features between MP-2 and FP-2A. Through a combination of structural modelling, enzymatic assays, molecular dynamics simulations, and virtual screening, our study elucidates the molecular and structural basis of substrate recognition in MP-2. These insights provide a foundation for selective, structure-based drug design targeting MP-2. Collectively, our findings establish MP-2 as a promising target for the development of selective inhibitors and contribute essential knowledge toward protease-selective antimalarial therapies.

## 2 Results

### 2.1 Structural and functional conservation of MP-2 with species-specific adaptations

We began our analysis of MP-2 by examining its domain architecture. Like other members of the C1 family of papain-like cysteine proteases (clan CA), MPs consist of an unstructured N-terminal pro-domain (InterPro: IPR013201) and a conserved C-terminal catalytic domain (InterPro: IPR000668). The pro-domain (residues 1–117) acts as an autoinhibitory segment, blocking the active site in the zymogen state. Activation occurs under acidic conditions in the parasite’s food vacuole, where the pro-domain is cleaved, enabling the mature enzyme to degrade haemoglobin.

**Fig. 1:**
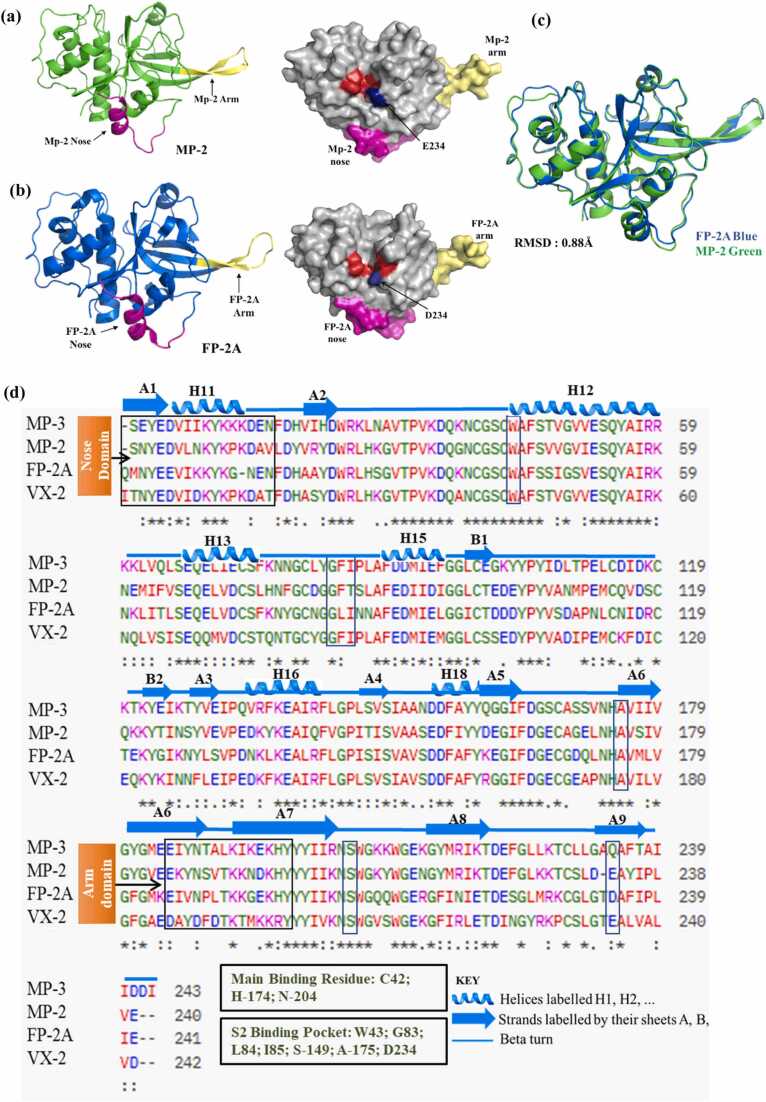
Structural and sequence comparison of MP-2 with FP-2A and related proteases. **(a)***Left panel:* Cartoon representation of MP-2 (green), highlighting the **nose domain** (involved in protein folding) in pink and the **arm/haemoglobinase domain** in yellow. *Right panel*: Surface representation of MP-2 in grey, with the S2 substrate-binding pocket highlighted in red and residue E234 within the pocket shown in blue. The nose and arm domains are also indicated in pink and yellow, respectively. **(b)***Left panel:* Cartoon representation of FP-2A (blue), with the nose and arm domains similarly highlighted in pink and yellow. *Right panel*: Surface representation of MP-2 (grey) again showing the S2 pocket in red and residue D234 in blue. Compared to FP-2A, MP-2 features a shallower substrate-binding cleft due to the D234E substitution, resulting in a more compact S2 cavity. This structural alteration enables MP-2 to accommodate both hydrophobic and charged P2 residues, whereas FP-2A, with a deeper cleft, interacts minimally with charged P2 residues (e.g., Arg or Lys). **(c)** Structural superposition of MP-2 (green) and FP-2A (blue) reveals a root-mean-square deviation (RMSD) of 0.88 Å, indicating high overall structural similarity, except for differences in the substrate-binding cleft. **(d)** Multiple sequence alignment (MSA) of MP-2, MP-3, FP-2A, and VX-2, annotated with secondary structural elements. α-helices (H1, H2…) and β-strands (A1, B1…) are marked above the sequences. The alignment highlights conserved features such as the nose and arm domains, as well as key residues forming the S2 pocket.

Sequence and phylogenetic analyses reveal a high degree of similarity between MPs and FP-2A, particularly within the C-terminal region (residues 244–484), which is critical for substrate binding and enzymatic stability. The sequence identity between MP2 and FP-2A was 60.38 %. Models of MP-2, MP-3, MP-4 and VX-2 were generated using Alphafold colab2. The crystal structure of FP-2A (PDB ID: 2GHU) was used for analysis. Structural alignments show that these isoforms adopt highly similar papain-like folds, with root mean square deviations (RMSD) under 1 Å, the aligned region included 239Cα atoms confirming strong structural conservation (Fig. 1; Supplementary Figure S1). Despite comparable molecular weights, MP isoforms display distinct isoelectric points (pI), attributed to differences in amino acid composition. MP-3 exhibits the highest pI (5.9), whereas MP-2 has the lowest (4.6), primarily due to an increased number of glutamic acid residues. Notably, VX-2 differs from the other proteases with a lower molecular weight (~24 kDa) and an intermediate acid pI of 4.66; it also displays reduced hydrophobicity alongside a distinctive amino acid composition characterized by higher proportions of glutamic acid, valine, tyrosine, and cysteine residues (**Tables ST1 and ST2**).

Structural alignment of MP-2 and FP-2A reveals a high degree of similarity, with a root-mean-square deviation (RMSD) of 0.88 Å (Fig. 1**c**). Both enzymes exhibit the characteristic papain-like fold, comprising two domains designated as L (left) and R (right) (Fig. 1). Notably, FP-2A possesses two unique structural motifs absent in classical cysteine proteases such as papain, cruzain, and cathepsins but present in MP-2: the FP-2A nose (~15 residues) and the FP-2A Arm (~14 residues) (**Table ST2**). The FP-2A nose forms a "nose-like" projection connecting the L and R domains, facilitating proper folding and catalytic activity without the need for a pro-domain a feature uncommon among papain-family proteases. This motif is conserved among plasmodial proteases, underscoring its functional significance. The FP-2A Arm, on the other hand, is a protruding "arm-like" β-hairpin structure extending from the protease surface. This motif is critical for haemoglobin binding and hydrolysis. Experimental deletion of the FP-2A Arm significantly impairs the enzyme’s ability to interact with haemoglobin, while its activity against other substrates remains unaffected. This indicates that the FP-2A Arm functions as an exosite, mediating substrate specificity for haemoglobin. These distinctive structural features of FP-2A and MP-2, absent in human homologs, present promising targets for the development of selective antimalarial therapies.

Structural modelling reveals that MP-2 harbours analogous features: the FP-2A nose forms a helical structure, and the FP-2A Arm adopts a β-hairpin configuration. Sequence alignments (Fig. 1**d**) across MP-2, MP-3, MP-4, and FP-2A confirm the conservation of these motifs, substrate-binding residues, and secondary structure elements. WebLogo plots (Supplementary Figure S2a) further underscore the conservation of functional motifs, while Supplementary Figure S2b demonstrates evolutionary conservation across *Plasmodium* species, supporting a shared catalytic framework tailored for species-specific haemoglobin degradation. Despite structural conservation, the variation in isoelectric points suggests potential functional divergence driven by differential host adaptation and regulatory mechanisms.

MP-2 and FP-2A both contains L (left) and R (right) domains with an intervening catalytic cleft. The cleft is known for its catalytic triad which is highly conserved: FP-2A features Cys42, His174, and Asn204 (PDB: 2ghu) while the corresponding residues in MP-2 are Cys25, His159, and Asn175. These residues perform analogous roles: cysteine serves as the nucleophile, histidine stabilizes the transition state, and asparagine positions the catalytic dyad, underscoring functional equivalence between the enzymes. This Cys-His-Asn triad is characteristic of papain-like proteases. In papain, for instance, Cys25 and His159 form the catalytic dyad, with Asn175 contributing to the stabilization of the active site [35]. The conservation of this catalytic architecture across MP-2, FP-2A, and papain underscores a shared mechanistic strategy among these cysteine proteases. This structural and functional conservation highlights the evolutionary preservation of the catalytic mechanism within the papain-like protease family.

Detailed analysis of the substrate-binding cleft reveals both conserved and divergent features. In all three proteases, the cleft comprises three sub-pockets: S1, S2, and S3, with the S2 sub-pocket being most critical for substrate recognition. In MP-2, residue E234 forms a compact around S2 portion of the cavity capable of accommodating both hydrophobic (e.g., Leu) and charged (e.g., Arg, Lys) P2 residues via electrostatic interactions (Fig. 1**a; Table ST3**). In contrast, FP-2A possesses an aspartate in the corresponding position, resulting in a deeper and more hydrophobic S2 sub-pocket with reduced affinity for charged substrates (Fig. 1**b**). In VX-2, the S2 pocket contains a mixture of polar (e.g., N176, N178, E237) and hydrophobic residues (e.g., F87, I88), creating a versatile binding environment. This dual character may enable VX-2 to balance recognition of both hydrophobic and polar P2 residues, providing greater substrate flexibility compared to FP-2A but with less pronounced charge complementarity than MP-2. This D234E (equivalent to D496E) substitution in MP-2 alters the geometry and electrostatics of the S2 sub-pocket, potentially expanding its substrate repertoire. Sequence alignment further confirms conservation of S2 sub-pocket residues among MP-2, MP-3, MP-4, and FP-2A (Fig. 1**d**), suggesting functional preservation with species-specific adaptations.

### 2.2 Structural and functional implications of S2 sub-pocket variations of MP-2

Our modelling studies identified the S2 sub-pocket as the most pronounced structural difference between MP-2 and FP-2A. Previous studies have also shown that residue D234 in FP-2A is essential for substrate recognition [36]. Building on this insight, we conducted a detailed structural and experimental analysis of the S2 sub-pocket in MP-2 to understand its functional implications. In MP-2, residue E234 (equivalent to E496) (highlighted in blue) contributes to the formation of a wide yet compact S2 portion of binding cavity composed of residues W304, G346, F347, T348, S412, A438, and E496 (Fig. 1**a,** Figure S3 **and Table ST3**). The extended side chain of E234 enhances the pocket’s flexibility, enabling interactions with both hydrophobic (e.g., Leu) and positively charged (e.g., Arg, Lys) P2 residues through electrostatic and hydrogen-bonding interactions. In contrast, FP-2A (Fig. 1**b**) exhibits a narrower and more rigid S2 sub-pocket cantered around D234. The shorter side chain of D234 limits electrostatic adaptability, restricting the pocket’s interaction profile. The FP-2A S2 sub-pocket comprises residues G83, L84, I85, S149, L172, and D234 (PDB: 2ghu) (**Table ST3**), forming a more hydrophobic and sterically constrained environment. Supplementary Figure S3 highlights this contrast, illustrating a more hydrophilic and electrostatically versatile S2 portion of the cleft in MP-2 compared to the compact, hydrophobic pocket in FP-2A.

**Fig. 2:**
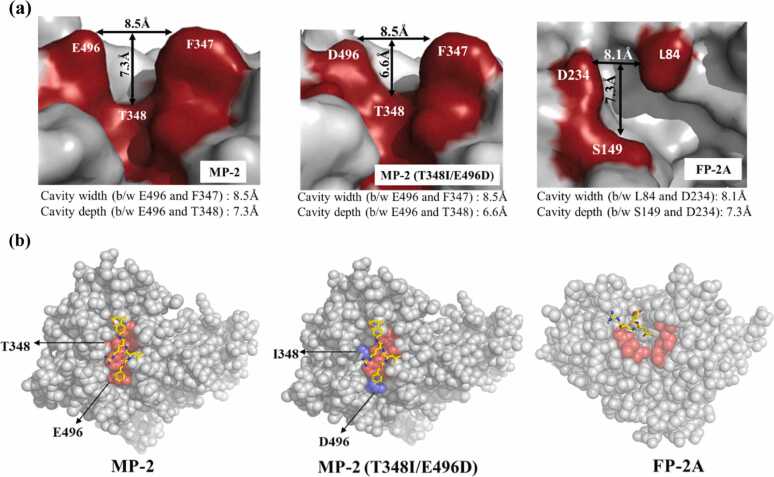
Structural analysis of the substrate-binding cleft in MP-2, MP-2 (T348I/E496D), and comparison with FP-2A. **(a)** Cavity property comparison of MP-2, MP-2 (T348I/E496D), and FP-2A. The cleft depth is reduced in MP-2 (T348I/E496D) due to the E496D mutation. In FP-2A, the cleft appears more constricted, partially attributed to the presence of D234 (equivalent to 496 residue position in MP-2). **(b)** Spherical representations of MP-2, MP-2 (T348I/E496D), and FP-2A, with the S2 pockets highlighted in red. WT MP-2 (T348I/E496D) are shown bound to the fluorogenic substrate Z-LR-AMC (yellow). In MP-2 (T348I/E496D), the substrate-binding cleft is notably shallower, primarily due to the E496D substitution. FP-2A is depicted bound to the covalent inhibitor E64 ((2S,3S)-trans-Epoxysuccinyl-L-leucylamido-3-methylbutane ethyl ester; yellow).

To explore the functional impact of these structural differences, we computationally engineered a series of MP-2 mutants, including several double and single mutants, designed to mimic features of the FP-2A S2 sub-pocket. Structural comparison of these mutants with wild-type (WT) MP-2 and FP-2A revealed that the modified MP-2 variants exhibited a shallower and more constricted S2 portion of the cleft (Fig. 2**a**). Despite these changes, the overall cleft volume remained comparable between MP-2 and FP-2A, with only subtle differences in width. WT MP-2 displayed cleft dimensions of 8.5 Å × 7.3 Å, which were reduced to 8.5 Å × 6.5 Å in the double mutant. In FP-2A, the cleft retained a similar depth but a narrower width (8.1 Å), a change attributed to the steric limitations (Fig. 2**b**). These observations suggest that substituting E496 with D (or equivalently E234D) in MP-2 shortens the side chain, thereby reducing pocket depth and altering substrate accommodation dynamics (**Table ST3**). To visualize these changes in substrate engagement, we modelled the binding of WT and mutant MP-2 to Z-Leu-Arg-AMC, a fluorogenic substrate that mimics haemoglobin-derived peptides (Figure S4). For FP-2A, the covalent complex with the inhibitor (2S,3S)-trans-epoxysuccinyl-L-leucylamido-3-methylbutane ethyl ester (E-64) was used for comparison (Figure S3a). These models highlighted distinct differences in cavity shape and electrostatics between the proteases.

**Fig. 3:**
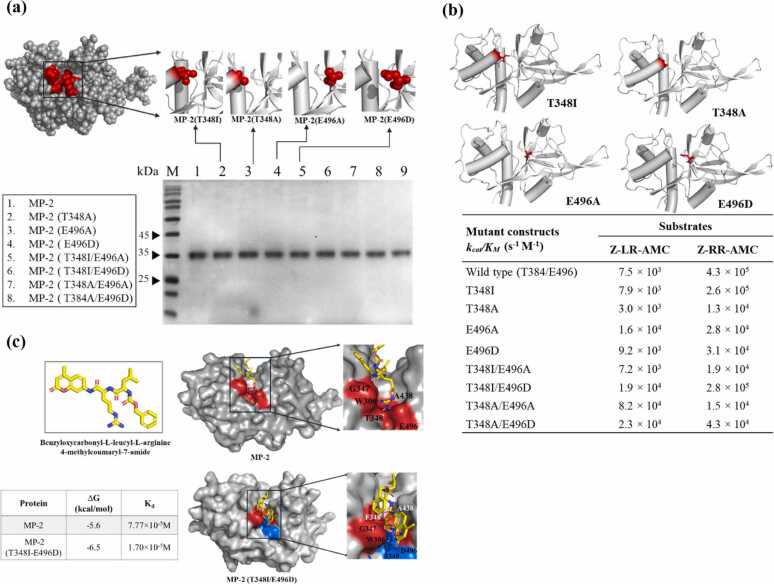
Structural and functional characterization of MP-2 mutants reveals the impact of site-specific mutations on enzymatic activity and substrate binding **(a)** SDS-PAGE analysis of purified wild-type and mutant MP-2 proteins, revealing single bands corresponding to comparable molecular weights. Top panel: structural models of the mutants (grey), with mutated residues highlighted in red. **(b)** Cartoon (cylindrical) representations of mutant variants of MP-2, with mutated residues shown in red. All variants were subjected to enzymatic activity assays using substrates Z-LR-AMC and Z-RR-AMC. The corresponding *k*_cat_/*K*_M_ (s^−1^ M^−1^) values are presented, demonstrating that even single- or double-point mutations can markedly influence substrate affinity and catalytic efficiency. **(c)** Computational docking studies validating substrate-binding alterations in mutant MP-2. The substrate Z-LR-AMC is shown in stick representation. A table summarizes the predicted binding free energies (ΔG, kcal/mol) for WT and mutant MP-2-substrate complexes. Surface representations depict the S2 pocket (in red) and mutated residues (in blue), illustrating structural rearrangements and changes in the binding microenvironment.

To experimentally validate our computational predictions, we expressed and purified nine MP-2 mutants, focusing on residues T348 and E496, which were identified as key components of the S2 sub-pocket. SDS-PAGE analysis confirmed the purity and monomeric nature of both WT and mutant proteins, with all showing a consistent molecular weight of ~36 kDa (Fig. 3**a; Table ST1**). Structural models highlighted the specific mutations in red, emphasizing their positions within the substrate-binding cleft. Thermal shift assays demonstrated that all mutants retained structural integrity, with only minor differences in thermal stability. The WT MP-2 exhibited slightly higher thermal stability (Tm ~ 68 °C) compared to most mutants, suggesting that the introduced mutations cause minor local perturbations rather than major conformational changes (Figure S5). The most pronounced reduction in thermal stability was observed in the E496D mutant (Tm ~ 58 °C). Interestingly, thermal stability was largely restored in the T348A/E496D double mutant (Tm ~ 65 °C), indicating a compensatory effect. These findings suggest that the mutations primarily alter substrate binding and pocket geometry without significantly disrupting the overall protein fold or structural integrity [28].

To evaluate the enzymatic efficiency of WT MP-2 and its mutant constructs, the kinetic parameters *k*_cat_ and *K*_M_ were determined using two fluorogenic peptide substrates, Benzyloxycarbonyl-_L_-leucyl-_L_-arginine 4-methylcoumaryl-7-amide (Z-LR-AMC) and Benzyloxycarbonyl-_L_-arginyl-_L_-arginine 4-methylcoumaryl-7-amide (Z-RR-AMC) (Figure S4**,** Table 1). The fluorescence emitted by the cleaved AMC was measured across a substrate concentration range of 0.1–100 μM. Michaelis constant *K*_M_, reflects the affinity of an enzyme toward its substrate; a lower *K*_M_ indicates higher substrate affinity. *k*_cat_, the turnover number, measures the catalytic efficiency of the enzyme; a higher *k*_cat_ corresponds to a faster conversion of substrate to product. The *k*_cat_/*K*_M_ ratio reflects overall enzymatic performance, with higher values indicating both higher affinity for the substrate and greater catalytic turnover. We studied detail Michaelis–Menten kinetics, allowing for the analysis of substrate affinity and turnover rates of WT MP-2 and its different mutants (Table 1). For Z-LR-AMC, the WT enzyme (T348/E496) exhibited a *k*_cat_ of 8.3 × 10⁵ s⁻¹ and a *K*_M_ of 111.2 µM. Among the mutants, T348A/E496A showed the highest *k*_cat_ of 1.8 × 10^7^ s⁻¹ and E496D showed the highest *K*_M_ of 923.9 µM. The lowest *k*_cat_ value 2.3 × 10^5^ s⁻¹ and *K*_M_ value 77.9 µM was exhibited by mutant T348A. For Z-RR-AMC, the WT enzyme (T348/E496) exhibited the highest *k*_cat_ value of 3.6 × 10^8^ s⁻¹ and a *K*_M_ of 846.4 µM. Among all mutants T348A/E496A exhibited the lowest *k*_cat_ value 2.2 × 10^6^ s⁻¹ and *K*_M_ value 143.1 µM (Table 1). Substrate hydrolysis kinetics for cysteine proteases VX-2 and FP-2A have been previously reported [27], and these values were used as benchmarks to compare the catalytic efficiency of MP-2. The catalytic efficiency *k*_cat_/*K*_M_ values for Z-LR-AMC were 7.34 × 10^5^ s⁻¹ ·M⁻¹ for VX-2 and 1.06 × 10^5^ s⁻¹ ·M⁻¹ for FP-2A, indicating that VX-2 is considerably more efficient in hydrolysing this substrate. In our study, the corresponding *k*_cat_/*K*_M_ value for the MP-2 was determined to be 7.5 × 10^3^ s⁻¹ ·M⁻¹ , whereas its double mutant MP-2(T348I/E496D) exhibited a markedly higher catalytic efficiency of 1.9 × 10^4^ s⁻¹ ·M⁻¹ for the same substrate (Fig. 3**b**). These findings clearly indicate that MP-2 catalyses Z-LR-AMC with substantially lower efficiency compared to VX-2 and FP-2A; However, the double mutation significantly enhances its catalytic performance, bringing it closer to the catalytic behaviour observed for FP-2A.Typically, catalytic efficiencies in the range of 10⁴–10⁵ M⁻¹ ·s⁻¹ are characteristic of enzymes with moderate substrate affinity and efficient turnover, suggesting that the MP-2 double mutant attains an optimally balanced catalytic profile for Z-LR-AMC. Interestingly, when the substrate was changed to Z-RR-AMC, MP-2 displayed a markedly higher catalytic efficiency (*k*_cat_/*K*_M_ ≈ 4.3 × 10⁵ s⁻¹·M⁻¹). This enhanced activity is likely due to electrostatic complementarity between the doubly charged arginine residues and the negatively charged E496 side chain, which may stabilize substrate binding and facilitate catalytic turnover by lowering the activation energy barrier. When the same substrate (Z-RR-AMC) was hydrolysed by the MP-2 double mutant (T348I/E496D), it exhibited a slightly reduced catalytic efficiency of 2.8 × 10⁵ s⁻¹ ·M⁻¹ .

**Table 1:** Detail kinetic parameters of MP-2 and its different mutant constructs.

Mutant constructs	Tm	Z-LR-AMC			Z-RR-AMC		
		*k*_*cat*_ (s^−1^)	*K*_M_ (µM)	*k*_*cat*_/*K*_M_ (s^−1^ M^−1^)	*k*_*cat*_ (s^−1^)	*K*_M_ (µM)	*k*_*cat*_/*K*_M_ (s^−1^ M^−1^)
MP−2	68.3	8.3 × 10^5^ ± 4.1	111.2 ± 1.2	7.5 × 10^3^	3.6 × 10^8^ ± 1.5	846.4 ± 0.6	4.3 × 10^5^
MP−2 (T348I)	66.3	4.2 × 10^6^ ± 5.2	534 ± 1.9	7.9 × 10^3^	1.2 × 10^8^ ± 2.3	469.5 ± 1.2	2.6 × 10^5^
MP−2 (T348A)	63.9	2.3 × 10^5^ ± 1.9	77.9 ± 1.3	3.0 × 10^3^	5.8 × 10^6^ ± 2.1	448.4 ± 1.6	1.3 × 10^4^
MP−2 (E496A)	62.2	4.8 × 10^6^ ± 0.7	290.7 ± 2.1	1.6 × 10^4^	1.1 × 10^7^ ± 6.5	393.7 ± 1.7	2.8 × 10^4^
MP−2 (E496D)	58.8	8.5 × 10^6^ ± 1.8	923.9 ± 0.9	9.2 × 10^3^	9.1 × 10^6^ ± 3.3	289.1 ± 0.9	3.1 × 10^4^
MP−2 (T348I/E496A)	62.7	5.4 × 10^6^ ± 0.6	760.1 ± 1.2	7.2 × 10^3^	1.2 × 10^7^ ± 1.8	637.7 ± 4.9	1.9 × 10^4^
MP−2 (T348I/E496D)	63.3	4.0 × 10^6^ ± 1.8	201.2 ± 6.1	1.9 × 10^4^	1.9 × 10^8^ ± 5.4	674.4 ± 3.1	2.8 × 10^5^
MP−2 (T348A/E496A)	65.4	1.8 × 10^7^ ± 3.3	220.7 ± 1.1	8.2 × 10^4^	2.2 × 10^6^ ± 2.7	143.1 ± 0.4	1.5 × 10^4^
MP−2 (T348A/E496D)	62.2	6.5 × 10^6^ ± 0.7	277.6 ± 1.4	2.3 × 10^4^	7.5 × 10^6^ ± 1.6	173.4 ± 0.7	4.3 × 10^4^

This trend was consistent with our computational predictions, which suggested that these mutations would have significant effects on substrate binding and catalytic performance. The T348I mutation replaces a polar threonine with a bulkier, hydrophobic isoleucine, which alters the S2 sub-pocket, creating a more hydrophobic environment that better accommodates the substrate. This structural change likely enhances the alignment of the substrate within the active site, leading to tighter binding and increased catalytic efficiency. Simultaneously, the E496D mutation shortens the acidic side chain, reducing the electrostatic complementarity between the enzyme and the substrate. This reduction in electrostatic interaction effectively decreases the depth of the S2 sub-pocket, which could negatively impact the binding of highly charged substrates. Interestingly, this mutant also lost its selectivity for charged substrates over hydrophobic substrates, exhibiting a behaviour more similar to FP-2A. This suggests that, by rationally engineering just one or two residues, MP-2 can be converted into a version with catalytic properties resembling FP-2A. Other MP-2 mutants displayed catalytic efficiencies that fell within the range defined by the WT enzyme and the T348I/E496D mutant, with variations depending on the specific residues substituted (Fig. 3**b**) [40]. These findings further reinforce the potential of rational enzyme engineering to fine-tune substrate specificity and catalytic efficiency, paving the way for designing targeted interventions for malaria treatment.

These kinetic findings were consistently reproducible across experimental replicates, reinforcing the reliability of the observed trends. To validate these results, we performed substrate docking analyses (Fig. 3**c**), which revealed a higher binding free energy for the MP-2 mutant (ΔG ≈ –6.5 kcal/mol) compared to the WT enzyme (ΔG ≈ –6 kcal/mol), supporting the experimental observations. Surface electrostatic potential mapping further corroborated these findings by showing substantial remodelling of the S2 sub-pocket in the mutant. Specifically, the introduced mutations (highlighted in blue) disrupted the electrostatic landscape, altering the physicochemical environment required for optimal substrate binding. Conservation analysis also highlighted the evolutionary and functional significance of residues T348 and E496 (Figure S2), underscoring their critical role in maintaining an electrostatically balanced and catalytically competent active site. Additionally, a detailed ConSurf-based analysis of the S2 pocket across various Plasmodium species revealed high conservation, with tryptophan (W) identified as the most conserved residue (Figure S6).

### 2.3 Dynamic stability and flexibility of WT MP-2 and mutant MP-2 (T348I/E496D) in comparison to FP-2A

**Fig. 4:**
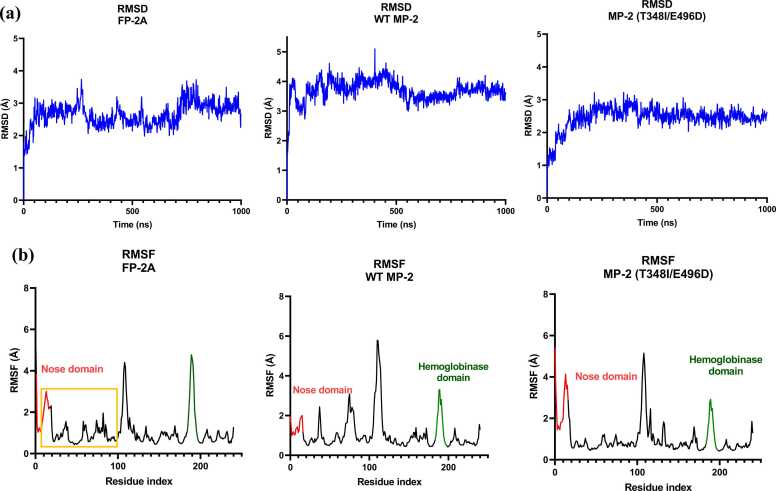
Molecular dynamics (MD) simulation analysis of MP-2, MP-2 (T348I/E496D), and FP-2A. **(a)** Root Mean Square Deviation (RMSD) plots of FP-2A, WT MP-2, and MP-2 (T348I/E496D) over a 1000 ns MD simulation. RMSD values (in Å) are plotted as a function of time (in ns) for FP-2A (left), MP-2 (middle), and MP-2 (T348I/E496D) (right). FP-2A exhibits moderate fluctuations, while MP-2 shows an initial rise in RMSD before stabilizing. The MP-2 (T348I/E496D) mutant displays slightly lower RMSD values. **(b)** Root Mean Square Fluctuation (RMSF) plots of residue-wise flexibility over the 1000 ns MD simulation. RMSF values (in Å) are plotted against residue indices for FP-2A (left), MP-2 (middle), and MP-2 (T348I/E496D) (right). The nose domain (red) shows the high fluctuations (~2–4 Å) across all three proteins, with a slight increase in mobility observed in the mutant. The haemoglobinase domain (green) also exhibits moderate flexibility; however, in the double mutant, a slight reduction in fluctuations suggests increased stabilization, likely due to cavity compaction induced by the mutations. The remaining regions of the proteins show relatively low fluctuations (~1–2 Å), indicating overall structural stability.

To complement our experimental and computational analyses, 1000-nanosecond molecular dynamics (MD) simulations were performed for WT MP-2, FP-2A, and the MP-2 double mutant (T348I/E496D) to evaluate their structural dynamics and flexibility **(**Figure S7**)**. To assess overall conformational stability, root mean square deviation (RMSD) plots were generated for each protein system (Fig. 4**a**). FP-2A exhibited moderate structural deviations (~2.8 Å) during the first 700 ns of simulation, followed by a slight increase in RMSD (~3.1 Å), which eventually stabilized by the end of the 1000 ns. This suggests the presence of two closely related conformational states near a global energy minimum, leading to minor dynamic shifts. In contrast, the WT MP-2 showed a higher initial RMSD (~4.5 Å), indicating significant structural rearrangement early in the simulation. The system stabilized after approximately 500 ns, suggesting that it had reached an energetically favourable conformation, maintaining a relatively stable trajectory thereafter. Notably, the MP-2 and FP-2A double mutant (T348I/E496D) displayed reduced conformational fluctuations compared to the WT, with RMSD values consistently ranging between ~2.5–3.0 Å and improved structural stability observed beyond 100 ns. These results indicate that the introduced mutations may contribute to enhanced structural rigidity or more stable folding of the protein.

To further dissect residue-level flexibility, root mean square fluctuation (RMSF) analyses were performed for all three proteins (Fig. 4b). Across all structures, fluctuations were observed in the N-terminal region, including the flexible nose domain (highlighted in red), with mutant MP-2 exhibiting the most pronounced variability (~4 Å). This behaviour is consistent with the inherent flexibility of this region, which is implicated in folding and functional dynamics (**Table ST2**: MP-2 nose domain, 16 residues, pI 5.84). Interestingly, in MP-2, dynamic fluctuations were predominantly localized to the nose domain, as evidenced by the absence of additional significant peaks next to this region. In contrast, both the WT MP-2 and the MP-2 double mutant exhibited at least two distinct peaks, suggesting the presence of additional flexible regions that may contribute to conformational transitions and functional dynamics in these proteins. Furthermore, in FP-2A, elevated fluctuations were also observed in a loop region located between β-strands B1 and B2 (Fig. 1**d**), a structural feature largely absent in MP-2 and its mutant. Notably, in the haemoglobinase domain, FP-2A displayed significant flexibility (~ 5 Å), whereas WT MP-2 showed reduced fluctuations (green; Table ST2: MP-2 haemoglobinase domain, 14 residues, pI 9.31). The mutant MP-2 exhibited a further decrease in this region (~3.5 Å), suggesting that the E496D mutation may enhance local compaction and stabilize the S2 substrate-binding sub-pocket.

We also analysed the solvent-accessible surface area (SASA) of all three proteins over the course of the 1000 ns simulation to evaluate conformational compactness and solvent exposure (Figure S8a). FP-2A consistently exhibited lower SASA values, fluctuating around ~11000 Å², suggesting a compact structure. In comparison, WT MP-2 showed slightly higher SASA values (~11,500–12,200 Å²), while the MP-2 double mutant (T348I/E496D) exhibited similar values with a minimal increase in SASA values (~11,900–12,500 Å²) Possibly due to a higher number of exposed N- and C-terminal residues (Figure S8a). Despite transient fluctuations, SASA remained largely stable across all trajectories, reflecting minimal large-scale conformational rearrangements and supporting the structural stability inferred from RMSD analysis.

To assess the global compactness and structural integrity of the systems, we calculated the radius of gyration (Rg) over the 1000 ns simulation period. FP-2A maintained a compact and stable profile, with Rg values tightly fluctuating around ~34.2 Å, consistent with its lower SASA and RMSD values. The WT MP-2 exhibited slightly broader Rg fluctuations (~34.0–34.8 Å), indicating comparatively higher conformational flexibility. Interestingly, the double mutant MP-2 (T348I/E496D) showed the highest Rg values (~35.0–36.0 Å) along with slightly greater variation, suggesting a more elongated conformation likely due to increased flexibility at the N- and C-termini, as also supported by the RMSF analysis. Overall, all three systems retained their global fold, though subtle differences in compactness and flexibility were evident (Figure S8b).

Hydrogen bond dynamics were analysed to assess the structural cohesion and internal stability of the three protein systems. FP-2A maintained a consistent number of hydrogen bonds (~12,400) throughout the simulation, suggesting stable intramolecular interactions. WT MP-2 showed a slightly higher hydrogen bond count (~12,600), with minor fluctuations indicative of flexible but stable regions. Notably, the MP-2 double mutant (T348I/E496D) exhibited the highest number of hydrogen bonds (~13,900), potentially indicating compensatory interactions introduced by the mutations that enhance the overall structural stability. This observation is further supported by the lower RMSD values, reinforcing the mutant’s stabilized conformation (Fig. 4). Overall, the hydrogen bonding patterns remained steady across all systems, supporting the notion of well-folded, dynamically stable proteins. (Figure S8c).

Collectively, these molecular dynamics results indicate that the T348I/E496D mutations in MP-2 do not compromise the structural core of the enzyme but modulate the dynamics of substrate recognition regions. This altered flexibility may influence substrate binding and has potential implications for inhibitor design and drug targeting.

## 3 Exploiting the therapeutic potential of MP-2 through selective inhibition

Given the observed differences in substrate recognition between FP-2A and MP-2, we hypothesized that these variations could be leveraged for species-selective antimalarial drug development. Such a strategy could potentially delay the emergence of drug resistance by minimizing cross-species selective pressure. To explore this, we employed a virtual drug screening platform designed to identify selective inhibitors targeting the S2 sub-pocket, thereby demonstrating the feasibility of species-specific therapeutic targeting.

**Fig. 5:**
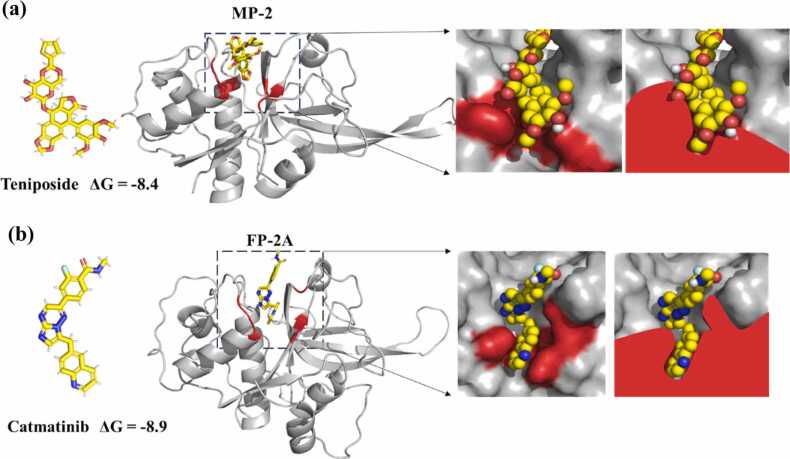
Selective drug targeting of the S2 sub-pocket in MP-2 and FP-2A. **(a)** Predicted binding mode of teniposide to MP-2. Virtual drug screening identified teniposide as a high-affinity, selective binder to the S2 sub-pocket of MP-2. For virtual screening purpose, the MTiOpenScreen drug library online server version (https://bioserv.rpbs.univ-paris-diderot.fr/services/MTiOpenScreen/) was used extensively, as it comprises a collection of purchasable, FDA-approved drugs. The left panel shows the chemical structure of teniposide. The center panel presents a cartoon representation of MP-2 bound to teniposide, with the S2 sub-pocket highlighted in red. The right panel offers a close-up surface view, illustrating how teniposide (yellow) fits within the S2 pocket and interacts with key residues in the binding cavity. **(b)** Predicted binding mode of capmatinib to FP-2A. Capmatinib was identified through virtual drug screening as a selective binder to the S2 sub-pocket of FP-2A. The left panel displays its chemical structure. The center panel shows a cartoon representation of FP-2A in complex with capmatinib, with the S2 sub-pocket highlighted in red. The right panel provides a close-up surface view of capmatinib (yellow) within the S2 pocket, emphasizing the diversity of chemical scaffolds capable of engaging this site.

From the screening results for MP-2, teniposide a glycosylated podophyllotoxin derivative emerged as a high-affinity binder (Fig. 5**a**). Its chemical structure enabled extensive hydrogen-bonding and π–π stacking interactions with residue E234 and surrounding S2 sub-pocket residues (**Table ST3**). Structural modelling and docking revealed teniposide (depicted in yellow) fitting snugly into the S2 sub-pocket (shown in red), with a predicted binding free energy (ΔG) of approximately –8.4 kcal/mol, corresponding to a dissociation constant (K_d_) of roughly 10^9^ nM range.

For FP-2A, capmatinib a quinoline-based, FDA-approved kinase inhibitor was identified as a selective binder (Fig. 5**b**). Capmatinib engaged residue D234 and other S2 sub-pocket constituents via hydrogen bonding and hydrophobic interactions, with a predicted ΔG of –8.9 kcal/mol. Although both compounds displayed comparable binding affinities, their distinct chemical scaffolds suggest differential interaction profiles shaped by the unique environments of their respective target pockets.

Surface representation of the MP-2 binding cleft revealed a mixed physicochemical landscape, comprising hydrophobic (blue) and electrostatic (pink) regions (Figure S9). This dual nature supports teniposide binding, particularly through its polar moieties. In contrast, FP-2A’s S2 sub-pocket exhibited a more uniformly hydrophobic character, favouring capmatinib interaction. Additionally, comparison of the S3 pockets further highlighted family-specific structural divergence: MP-2 features a compact and versatile cavity, while FP-2A harbours a deeper and more hydrophobic cleft (Figure S3), reinforcing differential binding preferences.

Supporting this, physicochemical analyses revealed differences in overall hydrophobicity and isoelectric points between MP-2 (hydrophobicity −0.385; pI 4.6) and FP-2A (hydrophobicity −0.395; pI 4.94) (**Table ST1**). Charge distribution in the haemoglobinase domains also varied, with MP-2 exhibiting a higher net positive charge (+2) compared to FP-2A (+1) (**Table ST2**). This additional charge likely enhances MP-2’s interaction with teniposide’s polar functional groups. Collectively, these findings validate the S2 sub-pocket as a druggable site and underscore the potential of exploiting subtle structural and electrostatic variations between related proteases to achieve species-selective inhibitor design.

## 4 Discussion

Cysteine proteases are integral to *Plasmodium* species’ survival, particularly in haemoglobin degradation during the erythrocytic stage, rendering them compelling targets for antimalarial drug development. While FPs in *P. falciparum* have been extensively characterized, their counterparts in other *Plasmodium* species, such as MPs in *P. malariae*, remain less explored. This study offers a comprehensive structural and functional analysis of MP-2 and FP-2A, shedding light on their biochemical properties and potential as therapeutic targets. Employing an integrative methodology including homology modelling, site-directed mutagenesis, enzymatic assays, molecular dynamics simulations, and virtual drug screening we demonstrate that MP-2 is structurally robust, functionally distinct, druggable protease underscoring its promise as a species-specific target for combating chronic *P. malariae* infections. However, it is important to acknowledge that our reliance on computational structural models, while highly informative, presents inherent limitations due to the absence of experimentally determined MP-2 crystal structures. Obtaining high-resolution crystal or cryo-EM structures would significantly enhance the accuracy of structural insights, validate predicted conformations, and better inform rational drug design efforts targeting MP-2.

Our structural analyses reveal that MP-2 adopts a canonical papain-like fold conserved among clan CA proteases, with high structural similarity to FP-2A (RMSD < 1 Å). This conservation extends to the catalytic triad and substrate-binding residues, underscoring a shared mechanistic framework for haemoglobin hydrolysis. However, despite the overall structural conservation, MP-2 exhibits unique physicochemical properties most notably a lower pI and altered S2 sub-pocket architecture suggesting species-specific functional adaptations potentially driven by divergent host environments.

Understanding proteases at the residue level is essential, as even single amino acid substitutions can profoundly affect substrate binding and inhibitor recognition, especially when these residues reside within the active-site pocket. Such fine-scale structural variations are fundamental to defining specificity in protease–ligand interactions. Singh et al. recently demonstrated that targeted substitutions in human stefin-A (e.g., Q55, Y56, and L73) enhanced its interaction with Falcipain-2 by improving hydrogen-bonding networks and van der Waals complementarity within the S2 sub-pocket [37]. These subtle alterations significantly increased inhibitory potency toward the parasite enzyme while reducing affinity for host cathepsins, underscoring how single-residue modifications can effectively modulate selectivity.

A key structural distinction between MP-2 and FP-2A lies within the S2 substrate-binding sub-pocket, which critically determines substrate specificity. In MP-2, the substitution of glutamic acid (E234) for aspartic acid (D234 in FP-2A) enlarges the S2 cavity and introduces greater electrostatic flexibility, likely enhancing MP-2’s capacity to accommodate both hydrophobic and positively charged residues at the P2 position, as reflected by its high catalytic efficiency for Z-LR-AMC. Beyond these residue-level determinants, *Plasmodium* papain-like cysteine proteases exhibit notable conformational adaptability, enabling accommodation of diverse host haemoglobins with varying sequence and structural features. Mutagenesis of key residues (T348 and E496) followed by kinetic assays confirmed the functional relevance of the S2 sub-pocket architecture, with the T348I/E496D double mutant showing a substantial reduction in catalytic efficiency due to steric and electrostatic constraints. Collectively, these observations illustrate how localized residue changes and domain plasticity cooperatively shape substrate specificity, providing a rational framework for designing selective inhibitors that target parasite proteases while sparing host homologs.

Our site directed mutagenesis and enzyme kinetics studies are strongly backed by molecular docking and molecular dynamics (MD) simulations, reinforcing the notion that MP-2’s S2 sub-pocket is both structurally dynamic and functionally adaptable. While the double mutant preserved the global fold of the WT enzyme, subtle alterations in local flexibility and electrostatic potential particularly around the haemoglobinase domain significantly influenced substrate binding and catalytic turnover. These nuanced shifts in substrate engagement highlight how minor sequence variations among *Plasmodium* cysteine proteases may confer species-specific substrate preferences, potentially reflecting evolutionary adaptations for optimized haemoglobin degradation in distinct host environments. MD simulations further elucidated the structural stability and conformational dynamics of MP-2 and FP-2A over 1000 ns trajectories. Although all systems maintained their global folds, residue-level analyses revealed notable differences in flexibility within substrate-binding regions. WT MP-2 exhibited different dynamic fluctuations in the nose and haemoglobinase domains compared to FP-2A, a feature that may underlie its broader substrate specificity. In contrast, the double mutant showed reduced flexibility in the S2 sub-pocket, suggesting that mutation-induced compaction may hinder efficient substrate accommodation and catalysis.

The drugability of MP-2 and FP-2A was explored through virtual screening, which revealed structurally distinct inhibitors that preferentially bind to each protease. Teniposide and capmatinib emerged as selective ligands for MP-2 and FP-2A, respectively, each exploiting the unique topography and electrostatics of their target’s S2 sub-pocket. These findings highlight the feasibility of species-selective inhibitor development, an important consideration in the context of emerging drug resistance. The physicochemical analyses further support this notion, as the unique electrostatic landscape of MP-2’s active site facilitates specific interactions with polar moieties present in teniposide, a feature not mirrored in FP-2A’s more hydrophobic S2 section of the cleft.

Together, these findings underscore the dual evolutionary themes of conservation and diversification among *Plasmodium* cysteine proteases. While the core structure and catalytic machinery are conserved, variations in the catalytic cleft, pIs, and pocket geometries enable species-specific tuning of substrate recognition and inhibitor binding. Importantly, our results establish MP-2 as a structurally stable and catalytically competent enzyme, with a druggable and selectively targetable S2 sub-pocket. These properties position MP-2 as a viable target for the rational design of *P. malariae*-specific inhibitors, which could complement existing therapies and help address persistent or chronic infections.

## 5 Conclusion

This study provides the first comprehensive structural and functional characterization of MP-2 in comparison to FP-2A, revealing critical insights into the biochemical specificity and therapeutic potential of MP-2. Despite sharing a conserved papain-like fold and catalytic machinery with FP-2A, MP-2 exhibits distinct physicochemical and dynamic features particularly within the S2 sub-pocket that underpin its broader substrate specificity and adaptability. Integrative analyses combining mutagenesis, enzymatic assays, molecular dynamics simulations, and virtual screening demonstrate that MP-2 is not only structurally stable and catalytically competent but also harbours a uniquely druggable active site. The identification of selective inhibitors exploiting MP-2’s electrostatic and spatial characteristics further supports its viability as a target for species-specific antimalarial strategies. Altogether, these findings highlight the evolutionary diversification of cysteine proteases among *Plasmodium* species and position MP-2 as a promising molecular target for the development of tailored therapeutics against chronic *P. malariae* infections.

## 6 Limitations and Future directions

While experimental structure determination remains the gold standard, it is often costly and time-consuming. Advances in protein modelling algorithms have made computational approaches attractive alternatives for generating reliable structural models. In this study, homology modelling validated against FP-2A’s structure provided valuable insights into MP-2, yet it may not fully capture the enzyme’s native conformation, highlighting the need for experimental structure determination to improve accuracy. Similarly, enzymatic assays using synthetic substrates may not fully reflect physiological conditions, emphasizing the importance of future studies employing haemoglobin-derived peptides.

Although virtual screening identified promising inhibitors, including anticancer drugs such as teniposide and capmatinib, these findings require rigorous *in vitro* and *in vivo* validation. The known cytotoxicity of these compounds underscores the necessity for developing safer, malaria-specific derivatives. Additionally, focusing exclusively on the S2 sub-pocket may overlook contributions from adjacent subsites (S1, S3), which could further refine inhibitor design. Considering the chronic and low-virulence nature of *P. malariae* infections, MP-2 inhibitors may be most effective as part of combination therapies in endemic regions. Field studies on clinical isolates and assessments of resistance potential will be essential to enable clinical translation. Collectively, these observations highlight both the opportunities and challenges of targeting parasite-specific proteases, and they establish a foundation for the rational design of safer, more selective antimalarial therapeutics.

## 7 Experimental Procedures

### 7.1 Materials

Most chemicals used in this study were obtained from Sigma-Aldrich (St. Louis, MO, USA), unless otherwise specified. Molecular biology reagents such as Site-Directed Mutagenesis Kit and the QuickChange Multi Site-Directed Mutagenesis Kit was purchase from Agilent (Santa Clara, CA, USA), Protein Thermal Shift Dye Kit was purchased from Thermo (Waltham, MA, USA), and other molecular biology reagents were procured from Qiagen (Hilden, Germany). All experiments were conducted using double-distilled water from a Milli-Q system.

### 7.2 Site directed mutagenesis

The MP-2-pQE9 wild-type plasmid was used as the parental DNA template for site-directed mutagenesis. Complementary primer pairs were designed to introduce the following substitutions: T348 to I348 or A348, and E496 to A496 or D496 (**Table ST4**). Single (T348I, T348A, E496A, and E496D) and double (T348I/E496A, T348I/E496D, T348A/E496A, and T348A/E496D) point mutations were generated using the QuickChange Site-Directed Mutagenesis Kit and the QuickChange Multi Site-Directed Mutagenesis Kit (Agilent, Santa Clara, CA, USA), following the manufacturers’ protocols. Briefly, single-point mutations were introduced by amplifying the parental plasmid with *Pfu* Turbo DNA polymerase for 16 cycles, while double mutations were introduced using the QuickChange Multi Enzyme Blend for 30 cycles. Following *DpnI* digestion to remove the methylated parental DNA, the mutated plasmids were transformed into *E. coli* XL1-Gold competent cells. All mutations were confirmed by DNA sequencing [42].

### 7.3 Expressions and purifications of wild type (WT) and mutant MP-2

WT MP-2 was expressed and purified following previously established protocols [34]. Verified mutant plasmids were subsequently transformed into *E. coli* M15 (pREP4) cells (Qiagen, Hilden, Germany) for recombinant protein expression. Each MP-2 mutant was expressed, purified, and refolded as described previously [34]. Briefly, recombinant protein expression was induced with 1 mM isopropyl-β-D-thiogalactopyranoside (IPTG) for 4 h at 37 °C with shaking (250 rpm) to ensure adequate aeration. Bacterial pellets were harvested and lysed overnight in 8 M urea lysis buffer. Recombinant proteins were purified using nickel–nitrilotriacetic acid (Ni–NTA) affinity chromatography (Qiagen), and purity was assessed by SDS–PAGE. Approximately 200 mg of each purified protein was refolded using an optimized refolding buffer and subsequently concentrated using Centriprep devices with a 10 kDa molecular weight cut-off (Merck Millipore, Burlington, MA, USA). Autocatalytic maturation of MP-2 into its active form was induced by adjusting the pH to 5.0 in the presence of 10 mM dithiothreitol (DTT), followed by incubation at 37 °C for 2 h.

### 7.4 Thermal stability assay

A thermal shift assay was conducted to evaluate effect of mutations on the thermal stability of MP-2 proteins. Thermal unfolding of WT MP-2 and its mutant forms was performed using Protein Thermal Shift Dye Kit (Thermo) with reporter 6-carboxyfluorescein (FAM). The thermal profile ranged from 25 °C to 100 °C with ramp rate of 0.05 °C/s. The fluorescent melt curve plots were analysed by Protein Thermal Shift™ Software v1.4 (Thermo).

### 7.5 Michaelis–Menten kinetics assay of WT and mutant proteases

Kinetic activity of protease was performed via hydrolysis of fluorogenic peptide substrates, including benzyloxy carbonyl-_L_-leucyl-_L_-arginine 4-methyl-coumaryl-7-amide (Z-LR-MCA) and Z-_L_-arginyl-_L_-arginine MCA (Z-RR-MCA) (Peptide International, Louisville, KY, USA). The enzyme (20 nM) was added to the assay buffer (10 nM of each peptide substrate, 10 mM DTT, 100 mM sodium acetate (pH 5.0)), and the release of fluorescence was detected at excitation and emission wavelengths of 355 nm and 460 nm, respectively, over 20 min at 37 °C using Fluoroskan Ascent FL (Thermo Fisher Scientific, Vantaa, Finland).

### 7.6 Structural modelling of proteins

Homology models of MP-2 (Gene ID: PmUG01_09024600; residues 264–503), MP-3 (PmUG01_09024700), and MP-4 (PmUG01_09024800) were generated using the SWISS-MODEL (https://swissmodel.expasy.org/) web server, based on the crystal structure of FP-2A (PDB ID: 1YVB) [38]. Additional models were produced using ColabFold v1.5.5 (https://colab.research.google.com/github/sokrypton /ColabFold/blob/main/ AlphaFold2.ipynb) and found to be indistinguishable from those generated by SWISS-MODEL. All models were refined using GalaxyRefine (https://galaxy.seoklab.org/cgi-bin/submit.cgi?type=REFINE) [39], [40]. Model quality was assessed via SAVES v6.0 (https://saves.mbi.ucla.edu/). Structural visualization, cavity depth/volume measurements, and electrostatic and hydrophobic analyses were conducted using PyMOL (https://pymol.org/2/). Multiple sequence alignments (MSA) of *Plasmodium* cysteine proteases were performed using MEGA (https://www.megasoftware.net/), and results were analysed for residue conservation with ConSurf (https://consurf.tau.ac.il/) and WebLogo (https://weblogo.berkeley.edu/logo.cgi). Active-site MSA for drug-binding analysis was conducted using MUSCLE, and phylogenetic MSA was performed with Clustal Omega (https://www.ebi.ac.uk/Tools/msa/clustalo/) [41], [42], [43], [44]. Functional domain annotation was done using Pfam (http://pfam.xfam.org/) and InterPro (https://www.ebi.ac.uk/interpro/) [45].

### 7.7 Structural mapping of mutation and docking analysis

For structural mapping of mutations, the three-dimensional structure of FP-2A (PDB ID: 2GHU) was obtained from the Protein Data Bank (https://www.rcsb.org/), and modelled structures were used for other proteins. Substrate cleft residues were identified using PDBsum (https://www.ebi.ac.uk/thornton-srv/databases/cgibin/pdbsum/GetPage.pl?pdbcode=index html) [46]. Conservation analysis for FP-2A and MP-2 was performed using ConSurf, while physicochemical properties, including charge, were calculated using ProtParam. Structural visualization, superimposition, and analysis of neighbouring residues, hydrophobicity, and electrostatic surface features were carried out using PyMOL [47]. Other Computational analyses, including solvent-accessible surface area (SASA), thermal stability, and hydrophobicity (solubility), were performed using GetArea, SCOOP, and SoDoPE, respectively. Protein-substrate docking was carried out using the HDOCK webserver using default parameters. Binding affinity (ΔG, kcal/mol) and dissociation constant (Kd, M) at 25 °C were calculated using PRODIGY (https://rascar.science.uu.nl/prodigy/). Biased substrate docking in the S2 binding pocket of WT and mutant MP-2, along with binding affinity (ΔG) calculations, was carried out using AutoDock Vina [48]. Structural visualization, superimposition, and analysis of neighbouring residues, hydrophobicity, and electrostatic surface features were carried out using PyMOL.

### 7.8 Molecular dynamics simulation

Molecular dynamics (MD) simulations were carried out using the Desmond module of Schrödinger (Release 2025–2) to investigate the structural dynamics and stability of MP-2, FP-2A, and mutant proteins. Protein structures were prepared using the Protein Preparation Wizard, including bond order assignment, protonation state optimization, and restrained minimization using the OPLS4 force field. Each system was solvated in an orthorhombic box with a 10 Å buffer using the TIP3P water model, and neutralized with counterions along with 0.15 M NaCl to mimic physiological conditions. The systems underwent energy minimization and equilibration using the default relaxation protocol, followed by a 1000 ns production run in the NPT ensemble at 300 K and 1 atm. Post-simulation analyses were performed using the Simulation Event Analysis module, including calculation of root mean square deviation (RMSD) and fluctuation (RMSF) for Cα atoms, solvent accessible surface area (SASA) to evaluate dynamic changes in solvent exposure, and radius of gyration (Rg) to monitor the compactness of the protein structures over time. These analyses enabled comparative evaluation of WT and mutant proteases, with particular focus on the structural dynamics of the active site and substrate-binding regions.

### 7.9 Virtual drug screening for FP-2A and MP-2

Molecular docking-based virtual screening of potential inhibitors for MP-2 and FP-2A was performed using the MTiOpenScreen platform (https://bioserv.rpbs.univ-paris-diderot.fr/services/MTiOpenScreen/) [49]. The protein structures of MP-2 and FP-2A were prepared by optimizing side chains, assigning bond orders, and removing water molecules. The binding site coordinates were defined around the S2 sub-pocket of each protease. The screening was conducted against the Drugs-lib library, which comprises a curated collection of purchasable, approved drug compounds. Docking was carried out using the default MTiOpenScreen parameters, and the top-scoring compounds were selected based on predicted binding affinity (ΔG values). The binding poses of selected hits were visualized to assess key interactions with the active site, particularly within the S2 sub-pocket, to evaluate their potential as selective inhibitors.

## Ethical Approval

Not applicable.

## Funding

SC research is supported by Ramalingaswami Fellowship (BT/RLF/Re-entry/09/2019) by Department of Biotechnology, DBT. SD is supported by the Institute Fellowship from BITS Hyd for JRF Fellowship. This study was also supported by the National Research Foundation of Korea (10.13039/100007431NRF) grant funded by the Korea government (10.13039/501100014188MSIT) (RS-2025–02413635).

## CRediT authorship contribution statement

**Sian D’silva:** Visualization, Validation, Software, Methodology, Investigation, Formal analysis, Data curation. **Hương Giang Lȇ:** Writing – review & editing, Visualization, Validation, Methodology, Investigation, Formal analysis, Data curation. **Soumyananda Chakraborti:** Writing – review & editing, Writing – original draft, Visualization, Validation, Supervision, Software, Resources, Project administration, Methodology, Investigation, Funding acquisition, Formal analysis, Conceptualization. **Byoung-Kuk Na:** Writing – review & editing, Supervision, Resources, Methodology, Funding acquisition, Data curation, Conceptualization.

## Consent to Participate

Not applicable.

## Consent to Publication

Not applicable.

## Author contribution

All authors contributed towards the implementation of the research. SC and BKN designed the study. HGL and SD collected and analysed the data. HGL and SD, SC assisted in methodology. SC, HGL, BKN, and SD wrote the manuscript. SC and SD edited the manuscript. All authors read and approved the final form of the manuscript.

## Author Contribution and Declaration Statement

All individuals listed as authors meet the authorship criteria established by the journal. All authors contributed significantly to the research and its implementation. Specifically, SC and BKN conceptualized and designed the study; HGL and SD were responsible for data collection and analysis; HGL, SD, and SC contributed to the development of the methodology. The manuscript was written by SC, HGL, BKN, and SD, and subsequently edited by SC and SD. All authors have read and approved the final version of the manuscript.

We also affirm that this work is original, has not been published elsewhere, and has been approved by all authors. Any potential conflicts of interest and sources of funding have been appropriately disclosed. Additionally, we used ChatGPT (an AI language model) solely for grammatical and typographical refinements; all scientific content is entirely our own.

## Supporting information availability

The Supporting Information includes various computational analyses, such as detailed structures of different MP isoforms and their comparison with FP-2A, conservation analysis, and thermal stability studies of different mutants; all data are freely available online

## Declaration of Competing Interest

The author(s) affirm that there are no conflicts of interest associated with the publication of this manuscript. The research was carried out independently, without any commercial or financial influences that could be perceived as potential conflicts

## Data Availability

Detail computational including structural analysis, experimental details including protocol for mutagenesis and activity assay details and other materials are available from the corresponding author upon reasonable request.

## Footnotes

Appendix A Supplementary data associated with this article can be found in the online version at doi:10.1016/j.csbj.2025.10.043.
