# Supplementary material for: Decoding substrate recognition in malapain-2 through structural and mutational insights
Source: Comput Struct Biotechnol J. 2025 Oct 24;27:4740–52. doi: 10.1016/j.csbj.2025.10.043 (PMC12617615; doi:10.1016/j.csbj.2025.10.043)
Supplement: Supplementary file 1 — Supplementary material [file mmc1.docx]

**Decoding Substrate Recognition in Malapain-2 through Structural and Mutational Insights**

Sian D’silva^1#^, Hương Giang Lê^2#^, Byoung-Kuk Na^2*^, Soumyananda Chakraborti^1*^

^1^Department of Biological Science, Birla Institute of Technology and Sciences-Pilani (Hyderabad campus), Hyderabad, India, ^2^Department of Parasitology and Tropical Medicine, and Institute of Medical Science, Gyeongsang National University College of Medicine, Jinju 52727, Korea

# Authors have equal contribution

*Corresponding authors

**Email**:

Soumyananda Chakraborti: [soumyananda.chakraborti@hyderabad.bits-pilani.ac.in](mailto:soumyananda.chakraborti@hyderabad.bits-pilani.ac.in); ORCID: 0000-0002-7384-690X.

Byoung-Kuk Na: [bkna@gnu.ac.kr](mailto:bkna@gnu.ac.kr); ORCID: 0000-0002-6734-1673

**Supplementary Figures**


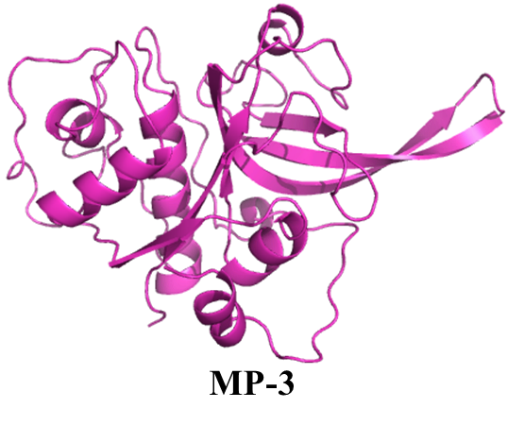

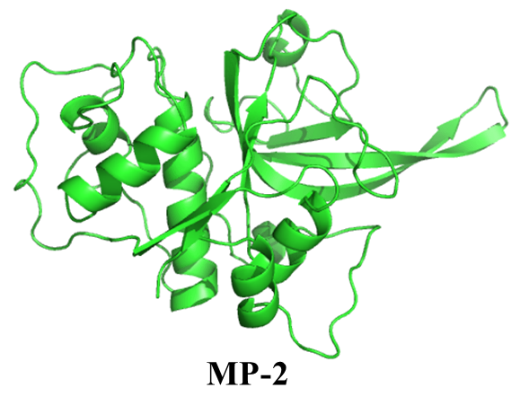


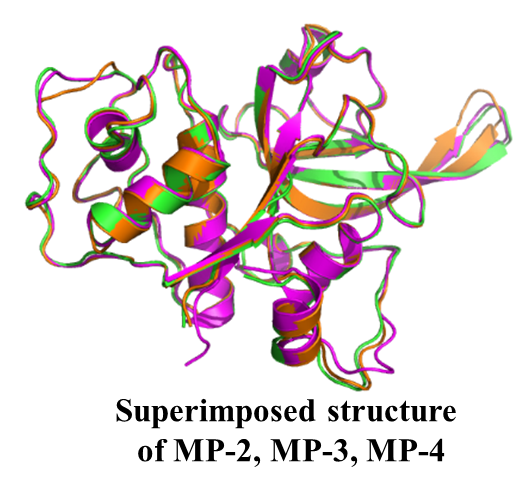


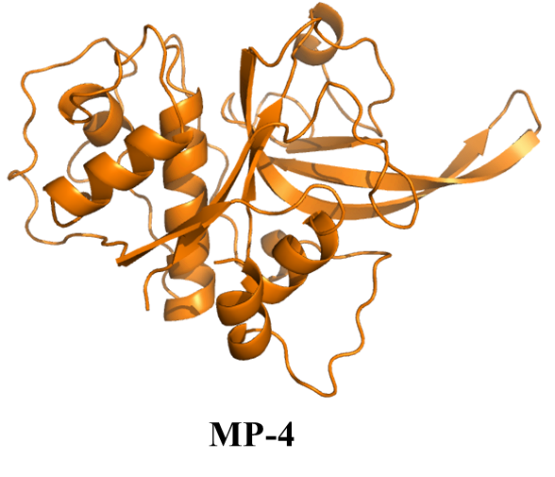


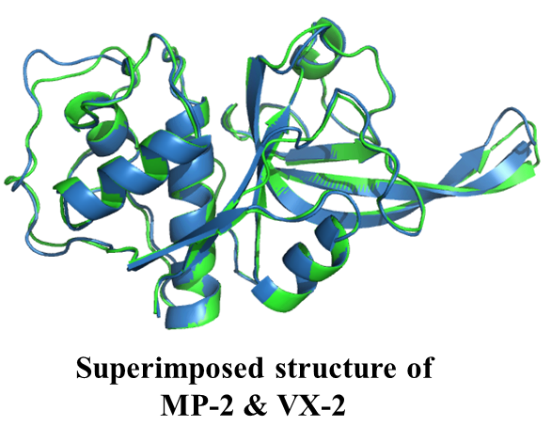


**
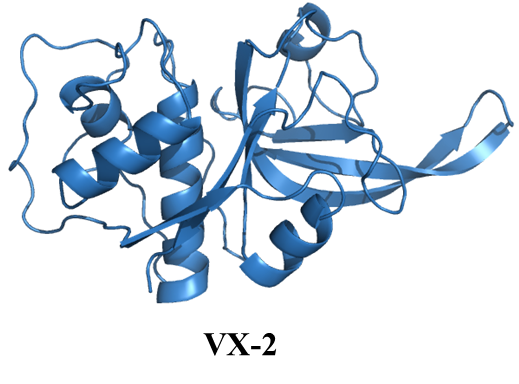
**

**RMSD = 0.85Å**

**Figure S1. Structural details of different *Plasmodium* cysteine proteases MP-2, MP-3, MP-4 and VX-2.** Cartoon representations of MP-2 (green), MP-3 (magenta), and MP-4 (orange) protein structures. The bottom right panel shows their superimposed alignment, with each structure highlighted in its respective color. The structural overlay reveals high overall similarity among MP-2, MP-3, and MP-4. The last panel depicts the cartoon representation of VX-2 (blue) followed by the superimposition of MP-2 (green), VX-2 (blue).

**
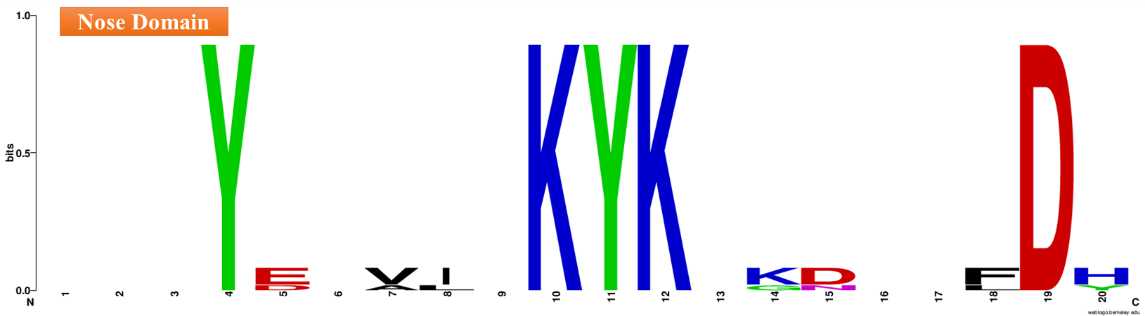
(a)**

**
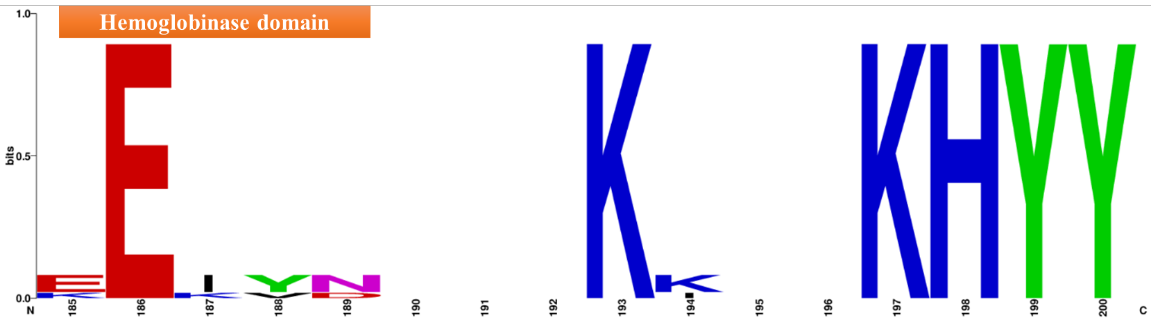
**

**
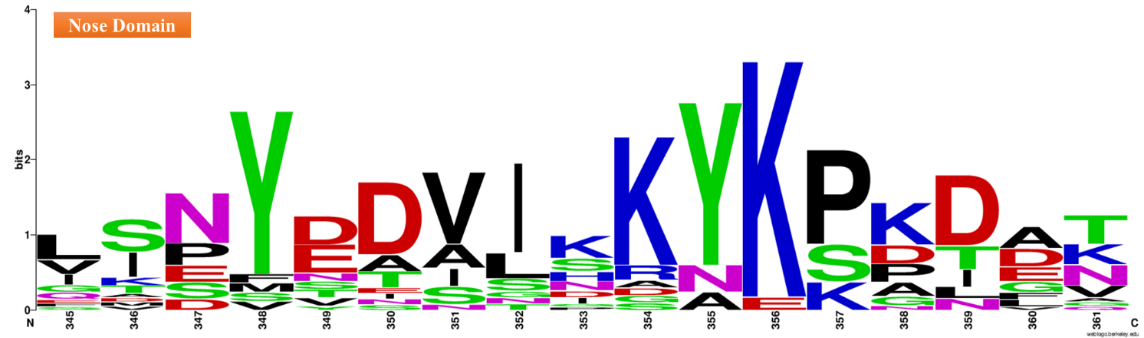
(b)**

**
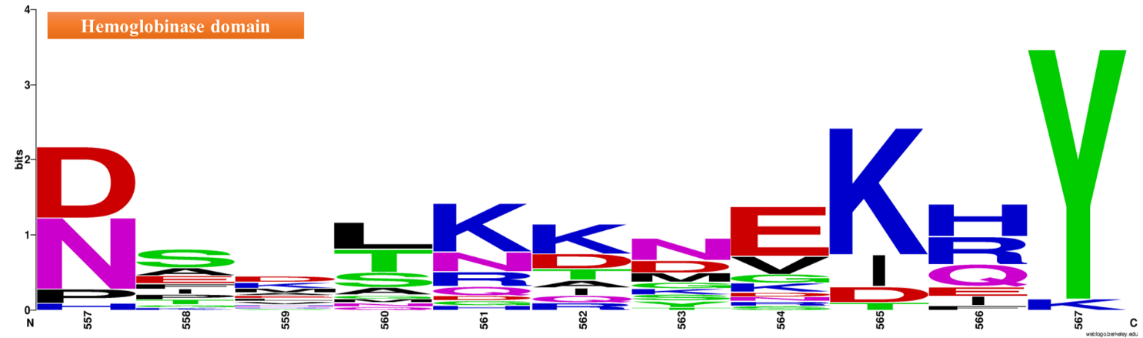
**

**Figure S2**. **Residue conservation across the nose and haemoglobinase domains based on WebLogo analysis**. (a) WebLogo plot showing conserved residues within the nose and haemoglobinase domains of FP-2A, MP-2, MP-3, and MP-4, highlighting shared sequence features across these proteases. (b) WebLogo plot illustrating conserved residues in the nose and haemoglobinase domains among cysteine proteases from various *Plasmodium* species (*P. knowlesi, P. vivax, P. ovale, P. chabaudi, P. reichenowi, P. berghei, P. yoelii, P. vinckei,* and *P. falciparum*), indicating evolutionary conservation within this protease family.

**
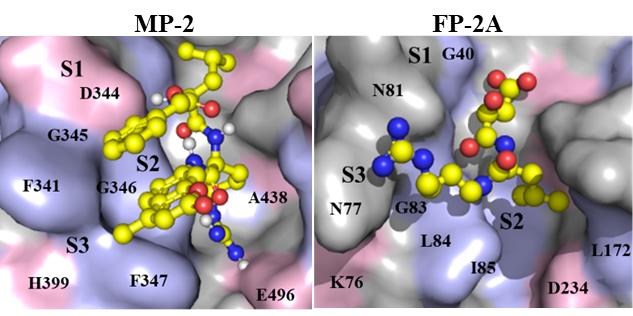
 (a)**

**
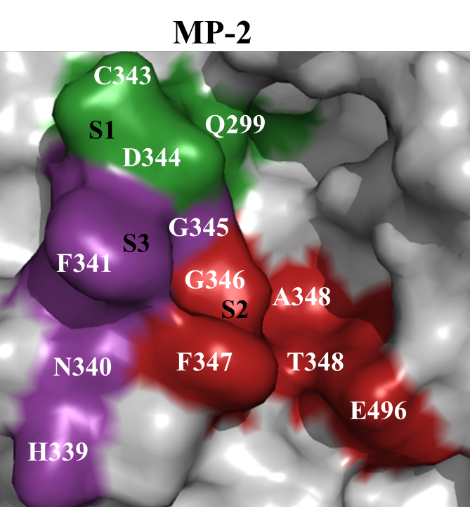

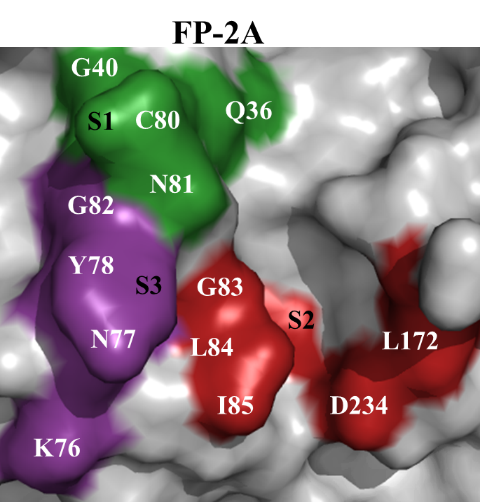
 (b)**

**Figure S3.**  Detailed surface representations of the substrate binding pockets of MP-2 (complexed with Z-Leu-Arg-AMC) and FP-2A (complexed with E64) are shown, highlighting the S1, S2, and S3 sub-pockets. Key hydrophobic (blue) and hydrophilic (pink) residues lining these regions are annotated to illustrate differences in the physicochemical landscapes and pocket geometries between MP-2 and FP-2A. In MP-2, the S1 (Q299, G303, C343, D344) and S3 (H339, N340, F341, G345) pockets feature a balanced distribution of hydrophobic and hydrophilic residues, promoting flexible substrate recognition. The S2 pocket (W304, G346, F347, T348, S412, A438, E496) in MP-2 is particularly diverse, combining aromatic, polar, and charged residues, which may contribute to substrate specificity and inhibitor binding. In contrast, FP-2A’s S1 (Q36, G40, C80, N81) and S3 (K76, N77, Y78, G82) pockets are primarily hydrophobic and polar-neutral, while the S2 pocket (G83, L84, I85, S149, L172, D234) comprises largely hydrophobic residues with a single acidic residue (D234), indicating a more constrained and hydrophobic binding environment. Residue details for each pocket are listed in Table ST3.

1. **(b)**

**
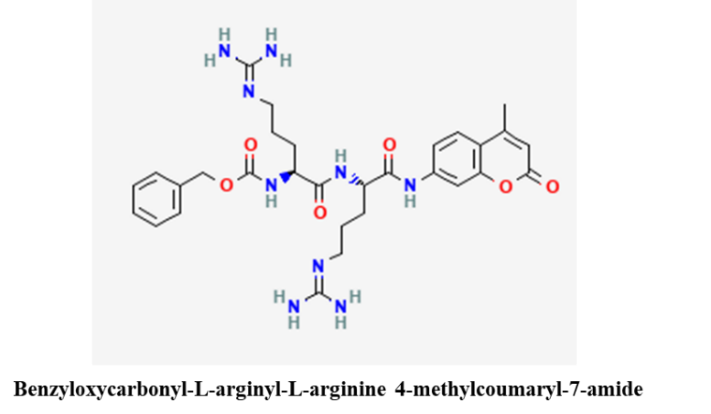

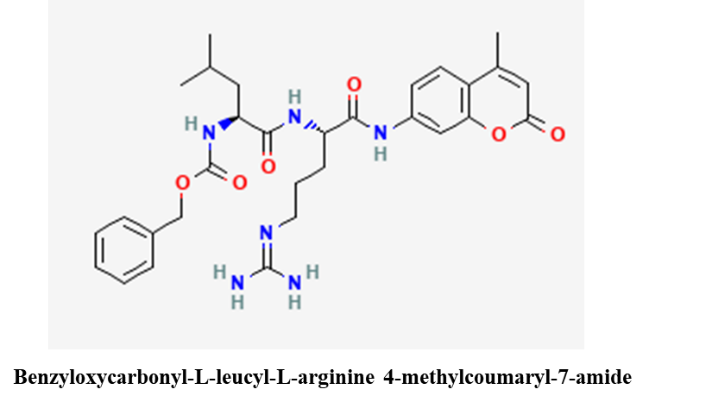
**

**Figure S4.** **Chemical structure of the two different substrates used in this study** (a) Benzyloxycarbonyl-_L_-leucyl-_L_-arginine 4-methylcoumaryl-7-amide (Z-LR-AMC) and (b) Benzyloxycarbonyl-_L_-arginyl-_L_-arginine 4-methylcoumaryl-7-amide (Z-RR-AMC).


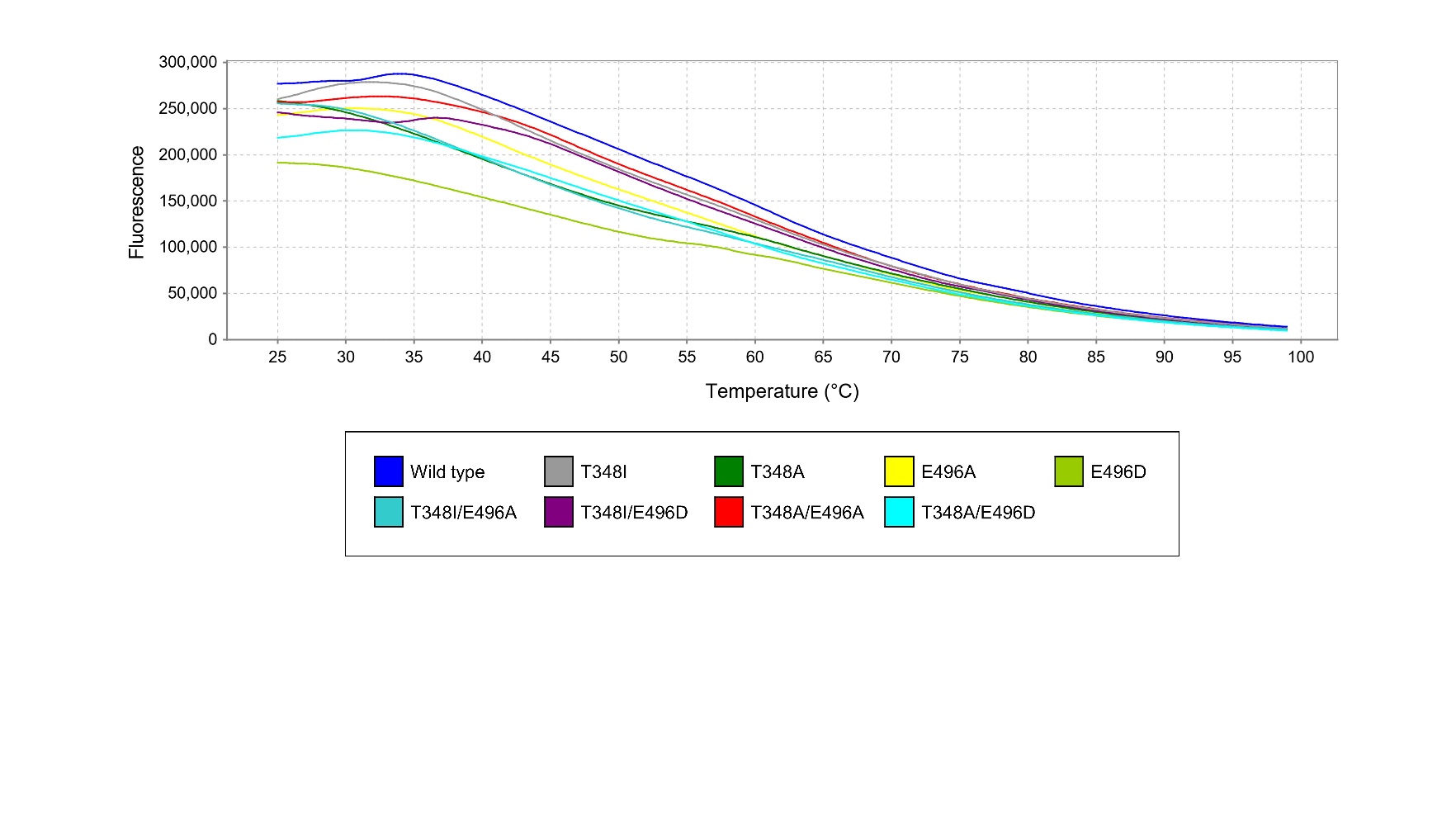


**Figure S5.**  **Thermal stability of wild-type MP2 and its mutant variants**. Thermal unfolding of wild-type MP2 and its various mutant forms was assessed by Thermal shift assay with reporter FAM from 25 °C to 100 °C. The wild-type protein (blue) exhibited higher fluorescence across all temperatures, indicating greater thermal stability compared to its mutants (T384I, T384A, E496A, E496D) and their respective combinations, as shown in the legend. Although the wild-type displays overall superior stability, the differences in thermal stability among the mutant variants are relatively modest based on the fluorescence data.

**
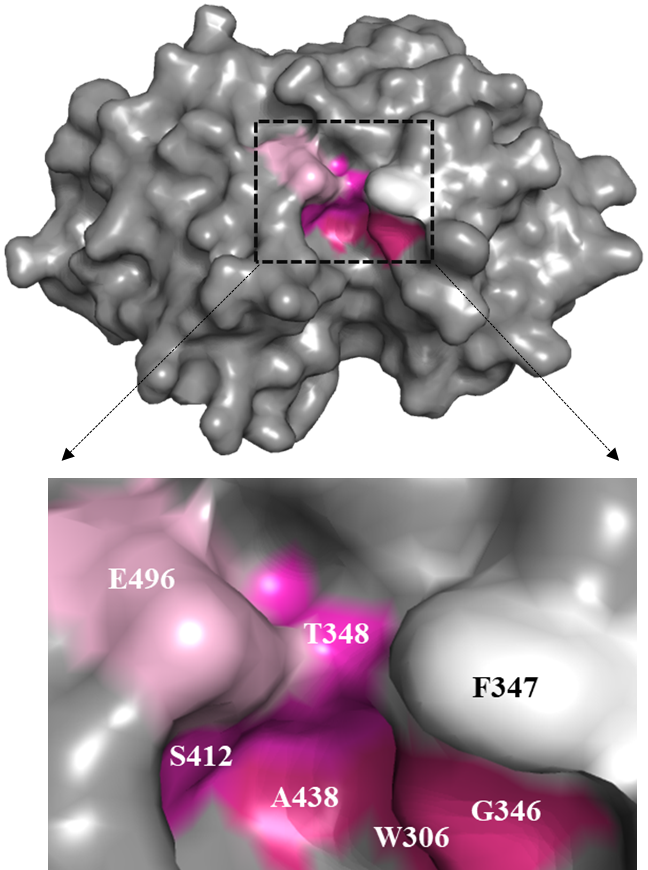
(a) (b)**


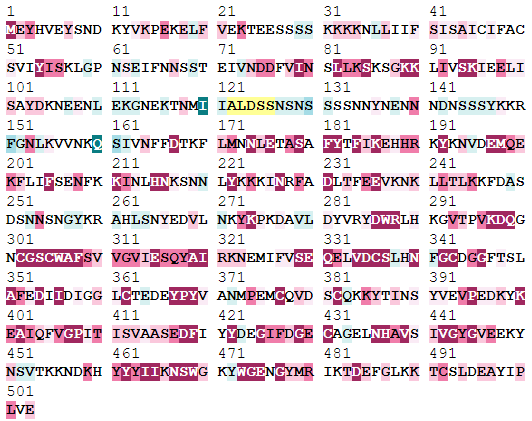


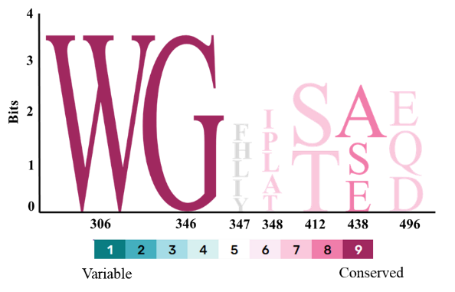
**(c)**

**Figure S6.** **Conservation analysis of the MP-2 S2 sub-pocket**. (a) Surface view of MP-2 with S2 pocket residues colored according to sequence conservation. (b) MP-2 sequence annotated based on conservation scores. (c) WebLogo plot illustrating conserved residues within the S2 sub-pocket across *Plasmodium* cysteine proteases (*P. knowlesi, P. vivax, P. ovale, P. chabaudi, P. reichenowi, P. berghei, P. yoelii, P. vinckei,* and *P. falciparum*).


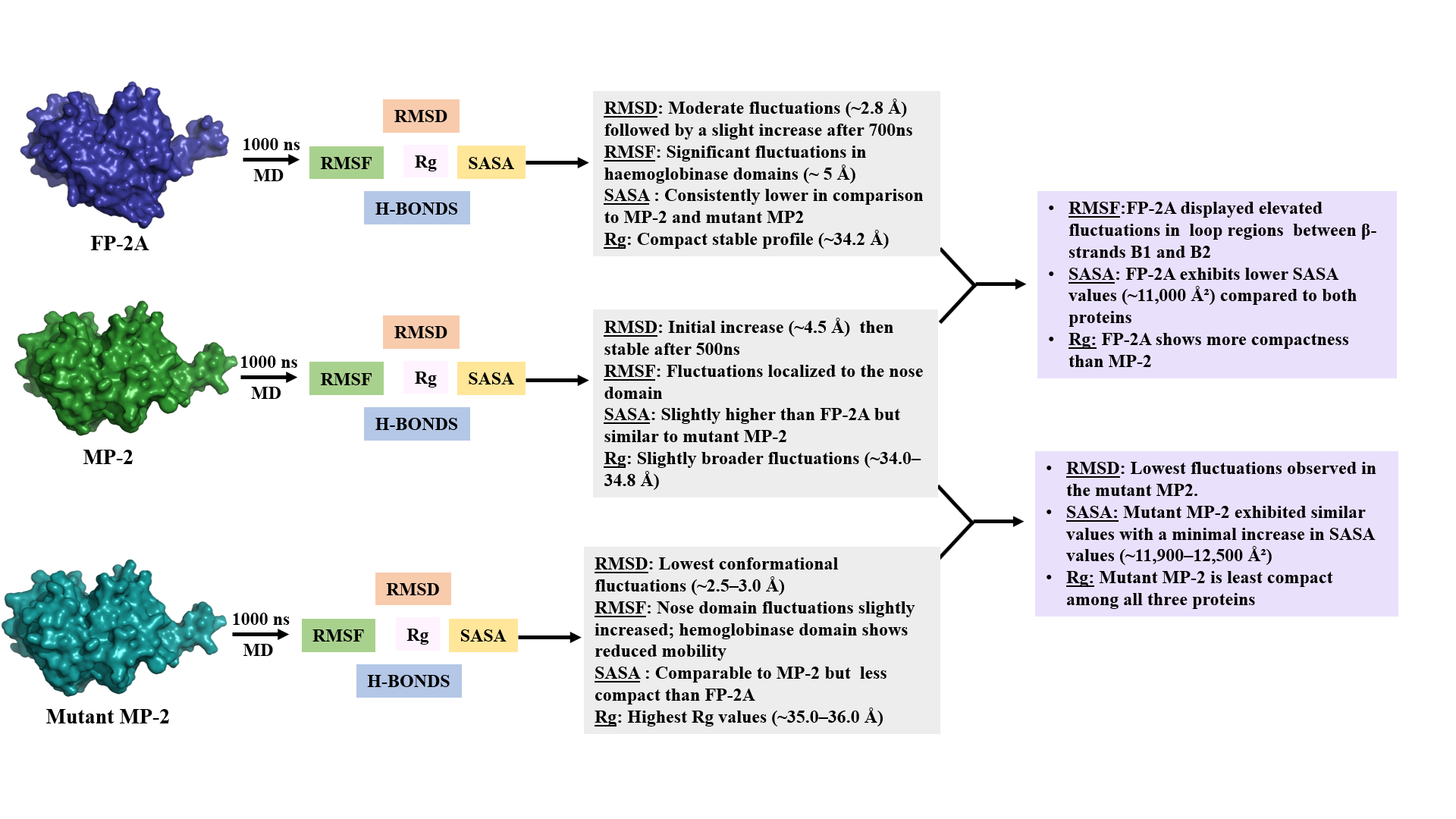


**Figure S7. Schematic overview of the molecular dynamics (MD) simulation setup and the resulting structural insights for FP-2A, MP-2, and the MP-2(T348I/E496D) mutant.**

**
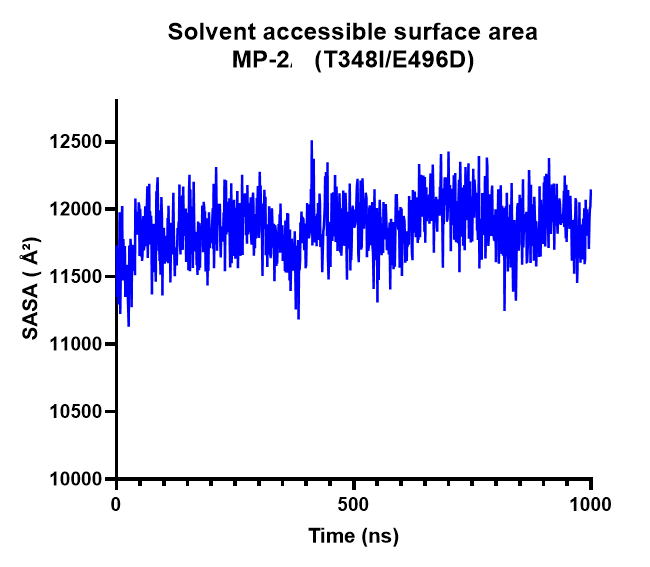

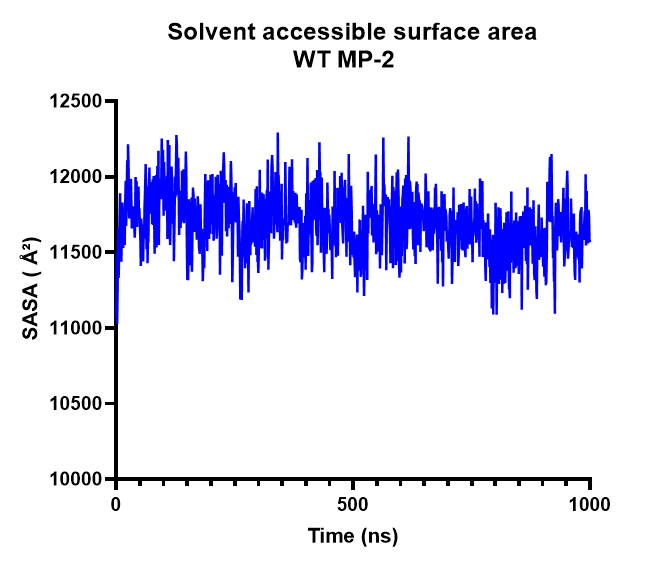
 (a)**

**
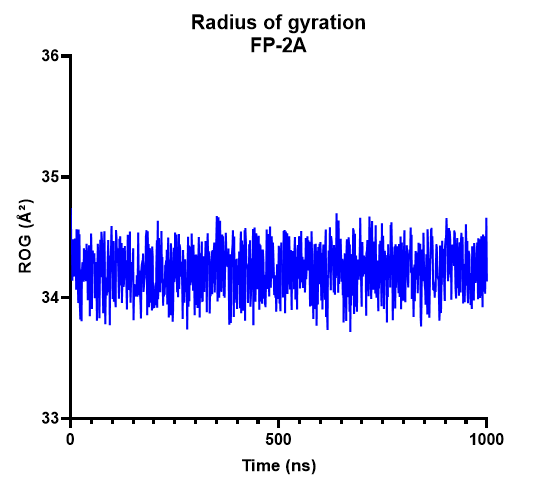
(b)**

**
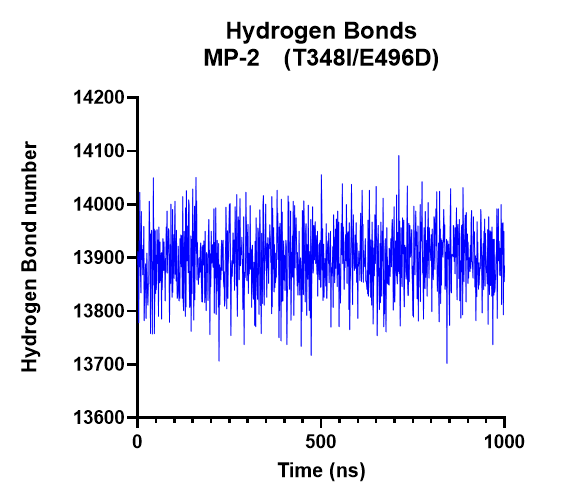

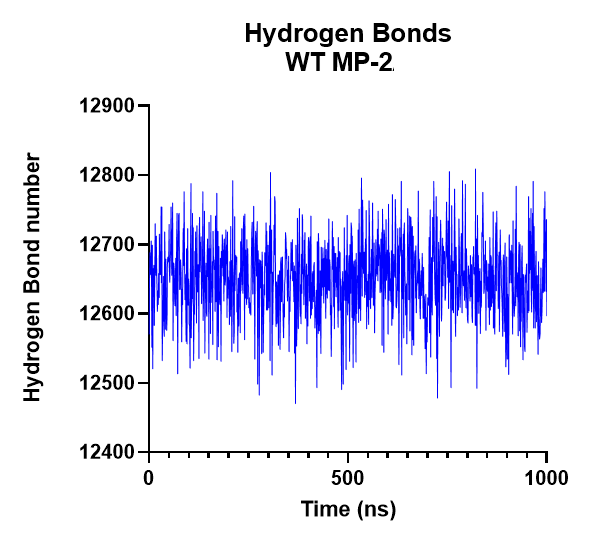
 (c)**

**]**

**Figure S8.** **Solvent Accessible Surface Area (SASA) and Radius of Gyration (Rg) Analysis of MP-2, MP-2 (T348I/E496D), and FP-2A Over 1000 ns of Simulation**. (a) Solvent Accessible Surface Area (SASA) profiles over a 1000 ns molecular dynamics simulation for FP-2A (left), WT MP2 (middle), and MP2 T348I/E496D mutant (right). All three systems exhibit relatively stable SASA values throughout the extended simulation period. Notably, FP-2A displays a consistently lower SASA compared to both WT MP-2 and the MP-2 mutant, suggesting a more compact or less solvent-exposed structure. The stability of SASA across the trajectories indicates minimal conformational changes during the simulation. (b) Radius of Gyration (Rg) plots over a 1000 ns molecular dynamics simulation for FP2A (left), WT MP-2 (middle), and MP-2 T348I/E496D mutant (right). The Rg values remain steady across all systems, with averages ranging between ~34–36 Å, indicating preserved overall structural compactness during the simulation. Although the mutant MP-2 (T348I/E496D) displays slightly higher fluctuations compared to WT MP-2 and FP-2A, these variations are modest and reflect only minor conformational flexibility. Overall, the consistent Rg values support the conclusion that the global structural integrity of these proteases remains stable over the extended simulation period. (c) Total number of hydrogen bonds over a 1000 ns molecular dynamics simulation for FP-2A (left), WT MP-2 (middle), and MP-2 T348I/E496D mutant (right). All three systems exhibit stable hydrogen bonding patterns throughout the simulation, indicating preserved structural cohesion. WT MP-2 shows a slightly higher average number of hydrogen bonds compared to FP-2A, while the MP-2 T348I/E496D mutant displays the highest hydrogen bond count, suggesting increased internal interactions or solvent engagement. The consistent hydrogen bonding profiles reflect stable intramolecular interactions over the course of the simulation.

**
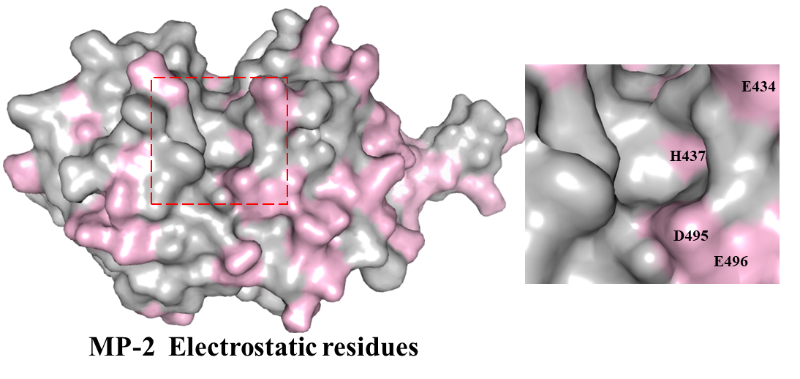
**

**
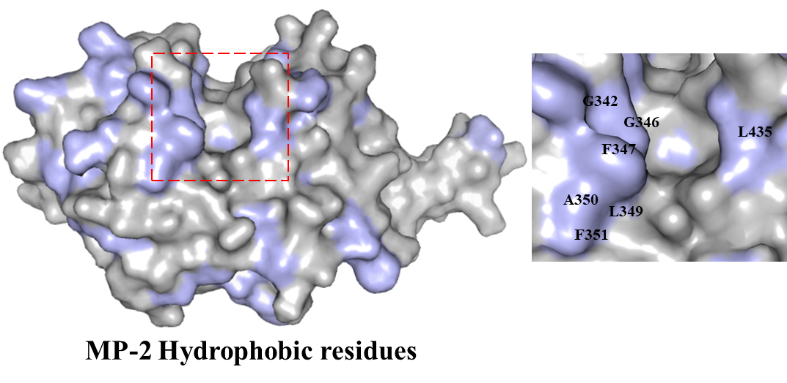
**

**Figure S9.**  **Surface representation** of MP-2, highlighting hydrophobic residues in blue and electrostatic residues in pink. A zoomed-in view emphasizes the substrate-binding cleft.

**Supplementary Tables**

**Table ST1**: Physicochemical properties of MP-2, MP-3, MP-4, FP-2A and VX-2, including molecular weight, calculated mass, hydrophobicity index, solubility profile, and composition of the first three N-terminal amino acids.

| **Protein Name** | **Mw** | **pI** | **Hydrophobicity** | **Amino Acid composition** | **Solubility** |
| --- | --- | --- | --- | --- | --- |
| MP-2 | ⁓27.5kDa | 4.6 | ‒0.385 | E, 9.2%; V, 8.8%; Y, 8.3%; C, 3.8% | Soluble |
| MP-3 | ⁓28kDa | 5.9 | ‒0.233 | I, 9.9%; K, 9.9%; E/G, 7.4%; C, 3.8% | Soluble |
| MP-4 | ⁓27kDa | 4.76 | ‒0.264 | G, 9.1%; I, 8.3%; K, 7.9%; C, 3.8% | Soluble |
| FP-2A | ⁓27kDa | 4.94 | ‒0.395 | G, 9.5%; K, 7.9%; I, 7.9%; C, 3.8% | Soluble |
| VX-2 | ⁓24kDa | 4.66 | ‒0.156 | G, 9.3%; E, 7.4%; V, 7.4%; C, 4.2% | Soluble |

**Table ST2:** Charge distribution and isoelectric points of the haemoglobinase and nose domains of FP-2A, MP-2, MP-3, and MP-4.

| **Protein Name** | **Haemoglobinase domain length** | **Total charge** | **pI*** | **Nose domain length** | **Total charge** | **pI** |
| --- | --- | --- | --- | --- | --- | --- |
| FP-2A | 14 a.a | ‒2 +3 = +1 | 8.5 | 15 a.a | ‒3 +3 = +0 | 6 |
| MP-3 | 14 a.a | ‒2 +3 = +1 | 8.48 | 16 a.a | ‒5 +4 = -1 | 4.93 |
| MP-2 | 14 a.a | ‒2 +4 = +2 | 9.31 | 16 a.a | ‒3 +3 = +0 | 5.84 |
| MP-4 | 14 a.a | ‒2 +4 = +2 | 9.31 | 16 a.a | ‒3 +3 = +0 | 6.12 |

*pI- Isoelectric point

**Table ST3:** Amino acid composition of the S1, S2, and S3 substrate-binding sub-pockets in FP-2A MP-2 and VX-2.

| **Protein Name** | **S1 pocket constituents** | **S2 pocket constituents** | **S3 pocket constituents** |
| --- | --- | --- | --- |
| FP-2A | Q36, G40, C80, N81 | G83, L84, I85, S149, L172, D234 | K76, N77, Y78, G82 |
| MP-2 | Q299, G303, C343, D344 | W304, G346, F347, T348, S412, A438, E496 | H339, N340, F341, G345 |
| VX-2 | Q39, C42, G43,C83, Y84 | F87, I88, S152,P175, N176,A178, E237 | Q79, N80, T81,G85, G86 |

**Table ST4. Primers used for MP-2 mutagenesis**

| Mutations | Sequences |
| --- | --- |
| MP-2(T348I) | T348I_F: 5’-GATGGTGGATTCA**T**CTCCTTAGCTTTTG -3’  T348I_R: 5’-CAAAAGCTAAGGAG**A**TGAATCCACCATC -3’ |
| MP-2(T348A) | T348A_F: 5’- GTGATGGTGGATTC**G**CCTCCTTAGCTTTTG -3’  T348A_R: 5’- CAAAAGCTAAGGAGG**C**GAATCCACCATCAC -3’ |
| MP-2(E496D) | E496D_F: 5’-CTTGCTCGTTAGATGA**C**GCGTATATTCCTTTAG -3’  E496D_R: 5’-CTAAAGGAATATACGC**G**TCATCTAACGAGCAAG -3’ |
| MP-2(E496A) | E496A_F: 5’-CTTGCTCGTTAGATG**C**AGCGTATATTCCTTT -3’  E496A_R: 5’-AAAGGAATATACGCT**G**CATCTAACGAGCAAG -3’ |
